# Germline Signals Deploy NHR-49 to Modulate Fatty-Acid β-Oxidation and Desaturation in Somatic Tissues of *C. elegans*

**DOI:** 10.1371/journal.pgen.1004829

**Published:** 2014-12-04

**Authors:** Ramesh Ratnappan, Francis R. G. Amrit, Shaw-Wen Chen, Hasreet Gill, Kyle Holden, Jordan Ward, Keith R. Yamamoto, Carissa P. Olsen, Arjumand Ghazi

**Affiliations:** 1 Department of Pediatrics, University of Pittsburgh School of Medicine, Pittsburgh, Pennsylvania, United States of America; 2 Division of Basic Sciences, Fred Hutchinson Cancer Research Center, Seattle, Washington, United States of America; 3 Department of Cellular and Molecular Pharmacology, University of California, San Francisco, San Francisco, California, United States of America; University of California San Francisco, United States of America

## Abstract

In *C. elegans,* removal of the germline extends lifespan significantly. We demonstrate that the nuclear hormone receptor, NHR-49, enables the response to this physiological change by increasing the expression of genes involved in mitochondrial β-oxidation and fatty-acid desaturation. The coordinated augmentation of these processes is critical for germline-less animals to maintain their lipid stores and to sustain *de novo* fat synthesis during adulthood. Following germline ablation, NHR-49 is up-regulated in somatic cells by the conserved longevity determinants DAF-16/FOXO and TCER-1/TCERG1. Accordingly, NHR-49 overexpression in fertile animals extends their lifespan modestly. In fertile adults, *nhr-49* expression is DAF-16/FOXO and TCER-1/TCERG1 independent although its depletion causes age-related lipid abnormalities. Our data provide molecular insights into how reproductive stimuli are integrated into global metabolic changes to alter the lifespan of the animal. They suggest that NHR-49 may facilitate the adaptation to loss of reproductive potential through synchronized enhancement of fatty-acid oxidation and desaturation, thus breaking down some fats ordained for reproduction and orchestrating a lipid profile conducive for somatic maintenance and longevity.

## Author Summary

Much is known about how increasing age impairs fertility but we know little about how reproduction influences rate of aging in animals. Studies in model organisms such as worms and flies have begun to shed light on this relationship. In worms, removing germ cells that give rise to sperm and oocytes extends lifespan, increases endurance and elevates fat. Fat metabolism and hormonal signals play major roles in this lifespan augmentation but the genetic mechanisms involved are poorly understood. We show that a gene, *nhr-49*, enhances worm lifespan following germ-cell removal. NHR-49 is increased in animals that lack germ cells by conserved longevity proteins, DAF-16 and TCER-1. NHR-49, in turn, increases levels of genes that help burn fat and convert saturated fats into unsaturated forms. Through synchronized enhancement of these processes, NHR-49 helps eliminate excess fat delegated for reproduction and converts lipids into forms that favor a long life. NHR-49 impacts these processes during aging in normal animals too, but using different regulatory mechanisms. Our data helps understand how normal lipid metabolic processes can be harnessed to adapt to physiological fluctuations brought on by changes in the reproductive status of animals.

## Introduction

Many studies have documented the apparent trade-off between aging and reproduction as reduced fertility is associated with increased lifespan in several species [1]–[3]. However, reproductive fitness also confers distinct physiological benefits [4], [5]. A growing body of evidence underscores the complex interactions between aging and reproduction [6]–[9] but the mechanisms underlying this dynamic relationship remain obscure.

Aging and reproduction are both inextricably connected to the energetics of fat metabolism. Reproduction is an energy-intensive process that relies heavily on lipid supplies and is influenced by lipid homeostasis. Epidemiological data indicate that obesity and low-body weight together account for ∼12% of female infertility [10]. Reproductive senescence in women and other female mammals is marked by re-organization of body fat and frequently associated with weight gain [11]. Similarly, obesity not only increases the susceptibility to a host of age-related diseases such as diabetes and CVD, it may also directly accelerate the aging clock by hastening telomere attrition [12]. Thus, it would appear that lipid metabolism influences both reproduction and the rate of aging and may provide the basis for the impact of these processes on each other. These molecular underpinnings are poorly understood and identifying them has relevance for multiple aspects of human health, procreation and longevity.

In recent years, the nematode *Caenorhabditis elegans* has provided unique insights into the effect of reproductive status on the rate of organismal aging [7]–[9]. In *C. elegans*, sperm and oocytes are generated from a population of totipotent, proliferating germline-stem cells (GSCs) whose removal increases lifespan and enhances stress resistance [13], [14]. This phenomenon is not just a peculiarity of a hermaphroditic worm, since similar lifespan extension is exhibited by *Drosophila melanogaster* and other insect and worm species following germline removal [15]–[17]. Moreover, ovarian transplantation experiments in mice [18] and studies in human populations [19] suggest that the reproductive control of lifespan may be widely prevalent in nature.

The longevity of germline-ablated *C. elegans* is entirely dependent upon the presence of the conserved, pro-longevity FOXO-family transcription factor, DAF-16 [13]. DAF-16 is part of a transcriptional network that is activated in intestinal cells when the germline is eliminated [20]. DAF-16 is a shared longevity determinant that increases lifespan in response to multiple stimuli, including reduced insulin/IGF1 signaling (IIS) [21]. On the other hand, TCER-1, the worm homolog of the conserved, human transcription elongation and splicing factor, TCERG1 [22], specifically promotes longevity associated with germline loss [23]. Other components of the intestinal transcriptional network include regulators of cellular processes such as autophagy (PHA-4, HLH-30) [24], [25], heat-shock response (HSF-1) [26], oxidative stress (SKN-1) [27] and transcriptional co-factors (SMK-1) [28]. In addition to these proteins, a steroid signaling cascade that includes the nuclear hormone receptor (NHR), DAF-12, and components of a lipophilic-hormonal pathway that synthesize the DAF-12-ligand, dafachronic acid (DA), enhance the lifespan of germline-ablated animals ([29]; reviewed in [7], [9]). DAF-12 mediates the up-regulation of another NHR, NHR-80, that is in turn required for the increased expression of fatty-acid desaturases that catalyze the conversion of stearic acid (SA, C18:0) to oleic acid (OA, C18:1n9) [30]. DAF-12 also promotes DAF-16 nuclear localization in intestinal cells following germline ablation [31]. Several lines of evidence suggest that DAF-16-mediated lifespan extension relies on modulation of fat metabolism, at least in part, and involves lipophilic signaling [32], [33]. However, the mechanism through which DAF-16 orchestrates these lipid-metabolic changes is not known. NHR-80 and DAF-16 function in parallel pathways and NHR-80-mediated SA-to-OA conversion is not sufficient to overcome the loss of DAF-16 [30]. Other lipid regulators, including NHRs, which may act in the DAF-16 pathway to alter fat metabolism following germline removal are yet to be identified.

DAF-12 and NHR-80 are two members of a family of ∼284 NHRs represented in the worm genome, most of which have been derived from a hepatocyte nuclear factor 4 alpha (HNF4α) ancestor [34]. Many NHRs are lipid-sensing factors that respond to fatty acid and steroid ligands to alter gene expression. One such factor, NHR-49, shows sequence similarity to HNF4α, but performs functions undertaken in vertebrates by peroxisome proliferator-activated receptor alpha (PPARα). PPARα is a member of the PPAR family of proteins which plays essential roles in vertebrate energy metabolism and it operates at the hub of a regulatory complex that impacts fatty-acid uptake, lipoprotein transport and mitochondrial- and peroxisomal β-oxidation [35]. In worms, NHR-49 regulates of mitochondrial- and peroxisomal β-oxidation and fatty-acid desaturation during development and under conditions of food scarcity [36], [37]. *nhr-49* mutants exhibit metabolic abnormalities, shortened lifespan and reduced survival upon nutrient deprivation [36], [37]. NHR-49 expression is also essential in a small group of GSCs that can survive long periods of starvation to re-populate the gonad and restore reproductive potential when the animal encounters food [38]. It is conceivable that this protein has a pervasive role in promoting organismal survival in diverse physiological contexts that induce metabolic flux and require the restoration of lipid homeostasis.

Despite the identification of several genes that encode lipid-modifying enzymes, how lipid homeostasis is re-established following germline loss, and how this translates into enhanced survival of the animal remains recondite. In this study, we identify a group of NHRs required for the longevity of germline-less *C. elegans*. We describe a role for one of these, NHR-49, in enhancing lifespan through modulation of specific lipid-metabolic pathways. We demonstrate that NHR-49 is transcriptionally up-regulated by DAF-16 and TCER-1 in the soma upon germline removal. NHR-49 causes the increased expression of multiple genes involved in fatty-acid β-oxidation and desaturation, triggering a metabolic shift towards lipid oxidation and an unsaturated fatty acid (UFA)-rich lipid profile. NHR-49 is critical for young germline-less adults to maintain their lipid reserves and *de novo* fat synthesis, and overexpression of the protein in fertile adults increases their lifespan modestly. *nhr-49* single mutants display similar biochemical and age-related lipid deficits but not the widespread reduction in β-oxidation genes’ expression seen in germline-less mutants. NHR-49 expression during normal aging is DAF-16 and TCER-1 independent. It is also dispensable for the lifespan extension mediated by reduced insulin/IGF1 signaling (IIS), a DAF-16-dependent longevity pathway, suggesting that the DAF-16- and TCER-1-directed elevation of NHR-49 is especially important for the metabolic and lifespan changes induced by germline loss. Our results suggest that through the concerted enhancement of fatty-acid oxidation and desaturation, NHR-49 may mediate the breakdown of fats designated for reproduction and restore lipid homeostasis. Together, they provide evidence for an important role for NHR-49 in adapting to loss of reproductive potential and augmenting longevity.

## Results

### NHRs that act in the *daf-16* and *tcer-1* pathway to promote longevity

Lipid signaling and fat metabolism play important roles in the reproductive control of aging [7]–[9]. Hence, to identify components of the DAF-16/TCER-1 pathway that confer lifespan extension upon germline loss, we focused on NHRs. These transcription factors are activated by lipid ligands and many of them modulate lipid-metabolic pathways. From the two large-scale, feeding RNAi libraries that cover a majority of the worm genome [39], [40], we derived a focused ‘NHR-library’ to perform an RNAi screen. Our ‘NHR-library’ included RNAi clones targeting 259 of the 283 worm NHRs. We used temperature-sensitive *glp-1* mutants, a widely used genetic model for the longevity resulting from germline removal [41]. Previously, we had identified a GFP reporter, *Pstdh-1/dod-8::GFP* that is jointly up-regulated by DAF-16 and TCER-1 in intestinal cells of long-lived *glp-1* mutants [23]. We used this strain (*glp-1;Pstdh-1/dod-8::GFP*) to screen our ‘NHR library’ for clones that prevented the up-regulation of GFP in young adults at 25°C.

**Figure 1:**
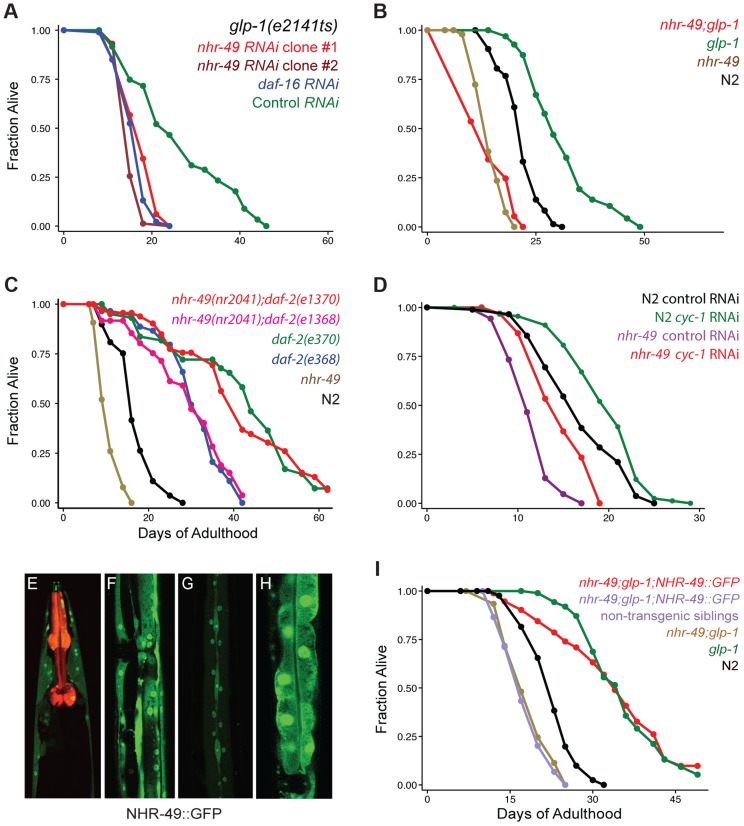
NHR-49 is essential for the longevity of germline-depleted animals and is widely expressed in somatic cells. **A: Effect of *nhr-49* RNAi on the lifespan of germline defective *glp-1* adults.** *glp-1* mutants were subjected to RNAi during adulthood by feeding bacteria containing control (empty) vector (green; m = 26.3±0.6, n = 92/96) as well as bacteria expressing dsRNA targeting *daf-16* (blue; m = 16.4±0.2, n = 92/97; P *vs.* control <0.0001), *nhr-49* RNAi clone #1 (red; m = 17.3±0.3, n = 99/101; P *vs.* control <0.0001, P *vs. daf-16* RNAi 0.01) and *nhr-49* RNAi clone #2 (maroon; m = 15.5±0.1, n = 79/92, P *vs.* control, <0.0001, P *vs. daf-16* RNAi 0.005). Clones #1 and #2 were obtained from the Ahringer and Vidal feeding RNAi libraries [39], [40], respectively. **B: Effect of** ***nhr-49*** **mutation on the lifespan of** ***glp-1*** **mutants and wild-type (N2) worms.** *glp-1* (green; m = 31.0±0.5, n = 94/101), *nhr-49;glp-1* (red; m = 14.1±0.1, n = 95/97; P *vs. glp-1*<0.0001), N2 (black; m = 21.6±0.1, n = 75/98), *nhr-49* (brown; m = 14.4±0.2, n = 89/100; P *vs.* N2<0.0001). **C: Effect of** ***nhr-49*** **mutation on the lifespan of** ***daf-2*** **mutants.** N2 (black; m = 16.9±0.1, n = 48/80), *nhr-49* (brown; m = 10.7±0.1, n = 80/91; P *vs.* N2<0.0001). *daf-2(e1368)* (blue; m = 30.6±0.4, n = 31/75; P *vs.* N2<0.0001), *nhr-49(nr2041);daf-2(e1368)* (pink; m = 29.5±0.7, n = 45/86; P *vs.* N2<0.0001; P *vs. daf-2(e1368)* 0.73). *daf-2(e1370)* (green; m = 43.0±0.6, n = 42/67; P *vs.* N2<0.0001), *nhr-49(nr2041);daf-2(e1370)* (red; m = 42.4±0.6, n = 46/74; P *vs.* N2<0.0001; P *vs. daf-2(e1370)* 0.004). **D: Effect of** ***cyc-1*** **RNAi on lifespan of N2 and** ***nhr-49*** **.** N2 worms grown on control vector bacteria (black; m = 16.9±0.3, n = 81/90) and on *cyc-1* RNAi bacteria (green; m = 19.6±0.3; n = 85/90; P *vs.* control <0.0002; percent increase in lifespan: 14). *nhr-49* mutants grown on control vector bacteria (purple; m = 11.6±0.1; n = 86/89) and on *cyc-1* RNAi bacteria (red; m = 14.8±0.2; n = 85/95; P *vs.* control <0.0001; percent increase in lifespan: 22**). E**–**H: NHR-49::GFP expression in adult somatic tissues.** NHR-49::GFP is visible in the cytoplasm and nuclei of neurons (E), muscle (F), hypodermis (G) and intestinal cells (H). *Pmyo-2::mCherry*, the co-injection marker, is seen as red fluorescence in the pharynx in E. I: **Rescue of the shortened lifespans of** ***nhr-49*** **and** ***nhr-49*** **;** ***glp-1*** **mutants by the NHR-49::GFP fusion protein.** N2 (black; m = 22.8±0.2, n = 81/92), *glp-1* (green; m = 36.3±0.2, n = 74/104, P *vs.* N2<0.0001), *nhr-49;glp-1* (brown; m = 17.9±0.2, n = 104/106, P *vs. glp-1*<0.0001), *nhr-49;glp-1;NHR-49::GFP* non-transgenic siblings (purple; m = 18.4±0.2, n = 101/107, P *vs. glp-1*<0.0001, P *vs. nhr-49*;*glp-1* 0.28), *nhr-49;glp-1;NHR-49::GFP* (red; m = 35.6±0.4, n = 58/102, P *vs. glp-1* 0.95, P *vs. nhr-49;glp-1*<0.0001, P *vs.* non-transgenic siblings <0.0001). All lifespan data are shown as mean lifespan in days (m) ± standard error of the mean (SEM). ‘n’ refers to the number of worms analyzed divided by total number of worms tested in the experiment (some worms were censored from the analysis as described in the methods section). P values were calculated using the log rank (Mantel Cox) method. Data from additional trials of these experiments are presented in S2 (panel A), S3 (panels B and I), S4 (panel C and D) Tables.

We identified 22 RNAi clones, targeting 19 *nhr* genes, which prevented *Pstdh-1::GFP* up-regulation. 16 of these clones (targeting 13 NHRs) reproducibly reduced GFP expression (S1 Table) and also shortened the extended lifespan of *glp-1* mutants, albeit with variable efficiency (11–48% suppression; S2 Table). We found that two independent RNAi clones targeting *nhr-49* completely abrogated the longevity of *glp-1* mutants (Fig. 1A, S2 Table). We chose to focus on *nhr-49* because of these strong phenotypes, and because it's functional similarity to PPARα provided an avenue for investigating the mechanisms that link fat metabolism and longevity.

### *nhr-49* is essential for lifespan extension of germline-ablated animals

To substantiate the *nhr-49* RNAi phenotype, we examined the effect of *nhr-49* mutation on the extended lifespan of *glp-1* mutants. We found that *nhr-49(nr2041),* a mutant that carries an 893 bp deletion, caused a suppression of *glp-1* longevity similar to that caused by *nhr-49* RNAi (Fig. 1B, S3 Table). The mutant also had a shorter lifespan compared to wild-type worms, as previously reported (Fig. 1B, S3 Table). Surprisingly, *nhr-49* was not essential for the longevity of *daf-2* mutants that live long due to impaired IIS and represent another DAF-16-dependent longevity pathway [21]. *nhr-49* mutation had no impact on the extended lifespan of *daf-2(e1368)* mutants in two of three independent trials and caused a small suppression in longevity in the third (Fig. 1C and S4A Table). Similarly, results were obtained with *nhr-49* mutants carrying another *daf-2* allele, *e1370*, (Fig. 1C and S4A Table) and upon RNAi-inactivation of *daf-2* in *nhr-49* mutants (S4B Table). In *C. elegans,* lifespan is also enhanced by perturbations to mitochondrial electron transport chain activity through a distinct regulatory pathway that is *daf-16* independent [42]. We found that RNAi treatment against *cco-1* and *cyc-1*, genes that encode components of mitochondrial electron transport chain, elicited a similar lifespan extension in *nhr-49* mutants as in wild-type worms (Fig. 1D, S4B Table). These observations suggest that *nhr-49* has variable degrees of relevance for different physiological alterations that influence aging. It is critical for the longevity mediated by reproductive signals but is not central to the lifespan changes resulting from reduced IIS or deficient mitochondrial electron transport.

### NHR-49 mRNA and protein levels are elevated in germline-less adults by DAF-16 and TCER-1

To address the role of *nhr-49* in the reproductive control of aging, we first examined NHR-49 localization in worms. We generated transgenic worms expressing GFP tagged to a full length NHR-49 transgene driven under control of its endogenous promoter from extra-chromosomal arrays (*Pnhr-49::nhr-49::gfp,* henceforth referred to as NHR-49::GFP). Animals expressing NHR-49::GFP showed widespread fluorescence throughout embryonic and larval development (S1A Figure). In adults, it was visible in all somatic tissues (Fig. 1E–H), localized to both nuclei and cytoplasm, with highest expression in intestinal cells (Fig. 1H). Expectedly, the transgene was silenced upon *nhr-49* RNAi except in neuronal cells (S1B Figure). To test if the NHR-49::GFP transgene was functional, we asked if it could rescue the shortened lifespan of *nhr-49*;*glp-1* double mutants. In two independent trials, NHR-49::GFP completely rescued the longevity of *nhr-49*;*glp-1* double mutants (Fig. 1I; S3 Table), whereas the rescue was 77% in a third trial (with strains generated by injecting the transgene at a lower concentration). This demonstrated that NHR-49::GFP is a functional protein that recapitulates the expression and function of the wild-type version.

**Figure 2:**
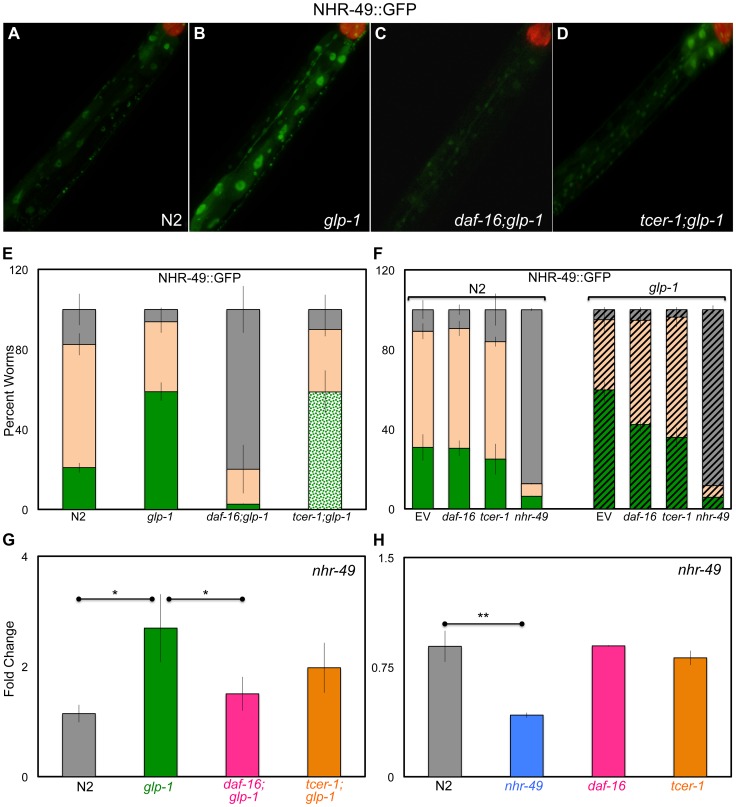
Germline removal causes increased expression of NHR-49 under regulation of DAF-16 and TCER-1. **A**–**E: Elevation of NHR-49::GFP in germline-less animals by DAF-16 and TCER-1.** NHR-49::GFP fluorescence observed in wild type (N) worms (A) (n = 219) and *glp-1* (B) (n = 383), *daf-16;glp-1* (C) (n = 175) and *tcer-1;glp-1* (D) (n = 267) mutants. The increased GFP in *glp-1* is visible in intestinal nuclei (compare A and B) and is abolished in *daf-16*;*glp-1* animals (C). *tcer-1*;*glp-1* mutants exhibit high expression in some gut cells but no GFP in others (D). The bar graph in E shows the quantification of these data obtained from day 2 young adults of each strain classified into those with high (green), medium (peach), and low (gray) GFP. In the *tcer-1*;*glp-1* bar, the high GFP class is shown as spotted green to indicate that these worms showed high but mosaic intestinal expression. **F: Selective effect of** ***daf-16*** **and** ***tcer-1*** **RNAi on NHR-49::GFP in** ***glp-1*** **mutants.** NHR-49::GFP fluorescence in wild-type animals (N2, solid bars) and in *glp-1* background (striped bars) observed in day 2 adults. Worms were grown on bacteria containing empty control vector (EV) or those expressing dsRNA targeting *daf-16, tcer-1* or *nhr-49*. GFP classification is the same as in E. *daf-16* or *tcer-1* RNAi treatments suppress the increased GFP seen in *glp-1* mutants, but not in wild-type worms (both strains were tested simultaneously). In the N2 background, n = 175, 113, 136 and 64, respectively for EV, *daf-16, tcer-1* or *nhr-49* RNAi, respectively. In the glp-1 background, n = 206, 146, 202 and 81, respectively for EV, *daf-16, tcer-1* or *nhr-49* RNAi, respectively. In E and F, ‘n’ signifies the total number of worms examined in three-to-five independent trials. **G, H: The control of** ***nhr-49*** **mRNA levels by DAF-16 and TCER-1 in fertile** ***vs.*** **germline-less adults.** Q-PCR analysis used to compare the mRNA levels of *nhr-49* between wild type (N2), *glp-1, daf-16;glp-1* and *tcer-1;glp-1* day 2 adults grown under similar conditions (G) as well as day 2 adults of N2, *nhr-49, daf-16* and *tcer-1* single mutants (H). Strains are represented on the X-axis and relative expression levels are on the Y-axis. The asterisks represent the statistical significance of the differences in expression in an unpaired, two-tailed t-test with P values 0.05 (*) and 0.005 (**). Error bars in E–H represent the standard error of the mean. In G, the difference between *glp-1* and *tcer-1;glp-1* was statistically significant in four of seven biological replicates (each with three technical replicates), but did not achieve significance when data from all the trials were combined.

Intestinal DAF-16 nuclear localization and TCER-1 transcriptional up-regulation are important molecular hallmarks associated with germline loss-dependent longevity [20], [23]. We asked if NHR-49 was similarly affected by germline removal. Germline depletion resulted in increased NHR-49::GFP, especially in intestinal cells (Fig. 2A, B, E, F). Next, we used the NHR-49::GFP reporter to test if this increased expression was dependent upon DAF-16 and/or TCER-1. In *glp-1* mutants carrying the *daf-16* null allele, *mu86*, GFP expression was dramatically and uniformly reduced in all tissues (Fig. 2C, E). *daf-16* knockdown by RNAi caused a similar but less marked reduction in NHR-49::GFP in *glp-1* mutants (Fig. 2F, striped bars). In *tcer-1*;*glp-1* double mutants, GFP expression pattern was unevenly affected. In most animals, some intestinal cells showed no GFP whereas others showed high GFP expression (Fig. 2D, E). It is not clear if the mosaic expression observed in *tcer-1*;*glp-1* mutants is a result of partial loss of function (the *tcer-1* allele, *tm1452,* is a 392 bp deletion coupled to a 10 bp insertion that is predicted to disrupt three of five transcripts produced by the gene) or reflects a spatial aspect of regulation by TCER-1. *glp-1* mutants subjected to *tcer-1* RNAi had reduced GFP expression as well (Fig. 2F, striped bars). In addition to these observations, an independent line of investigation supported the regulation of NHR-49 by DAF-16 and TCER-1. In an RNA-Sequencing (RNA-Seq) analysis designed to map the transcriptomes dictated by DAF-16 and TCER-1 upon germline ablation, we identified *nhr-49* as one of the genes jointly up-regulated by these two proteins (Amrit *et al.,* manuscript in preparation). Using Q-PCR assays, we confirmed that germline removal produced a significant increase in *nhr-49* mRNA, and this increase was repressed in *daf-16*;*glp-1* and *tcer-1*;*glp-1* mutants (the *tcer-1* mutant did not achieve statistical significance; Fig. 2G). Interestingly, DAF-16 and TCER-1 up-regulated *nhr-49* expression only in germline-ablated worms. In *daf-16* and *tcer-1* mutants alone, we did not observe a reduction in *nhr-49* mRNA during adulthood (Fig. 2H and S2 Figure). RNAi knockdown of these genes also did not reduce NHR-49::GFP levels (Fig. 2F, solid bars) suggesting that NHR-49 is differentially regulated depending on the reproductive status of the animal. Together, our experiments show that both mRNA and protein levels of NHR-49 are elevated in somatic cells upon germline removal. These changes are strongly dependent on DAF-16, at least partially dependent on TCER-1, and indicate that DAF-16 and TCER-1 mediate the transcriptional up-regulation of NHR-49 when the germline is eliminated.

### Somatic NHR-49 overexpression extends the lifespan of fertile worms

**Figure 3:**
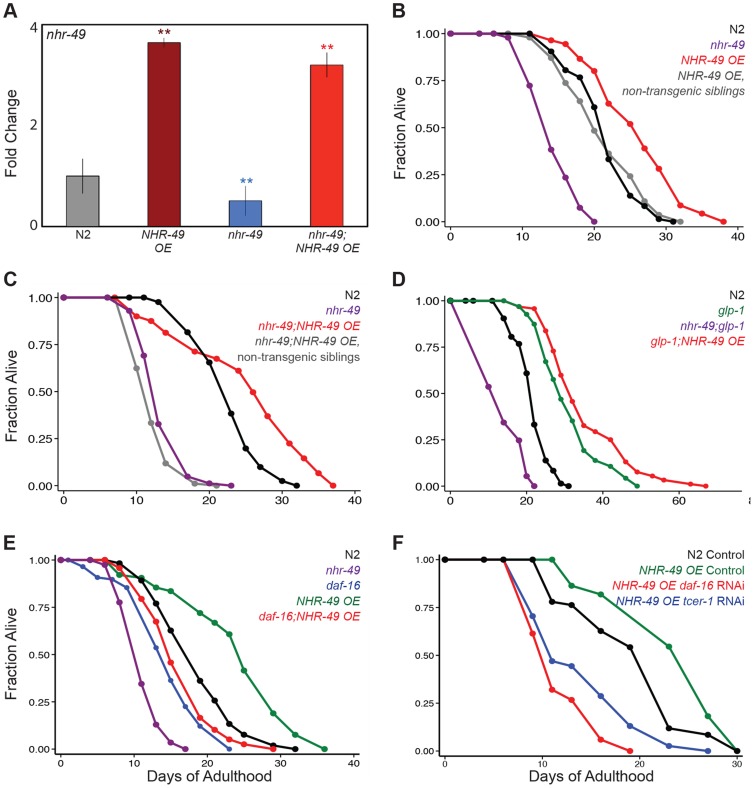
NHR-49 overexpression increases the lifespan of fertile worms. **A**: The levels of *nhr-49* mRNA compared using Q-PCRs between wild-type (N2, gray), *nhr-49* mutants (blue) and worms overexpressing NHR-49 through the NHR-49::GFP transgene (*NHR-49 OE*) in these two genetic backgrounds (maroon and red, respectively). The X-axis represents the strains being compared and the Y-axis the fold change in expression. The data is combined from four independent biological replicates, each with three technical replicates. Error bars display standard error of the mean, and asterisks depict the statistical significance of the differences observed in an unpaired, two-tailed t-test. N2 *vs. NHR-49 OE* P = 0.0006 (maroon asterisks); *nhr-49 vs. nhr-49*;*NHR-49 OE* P = 0.002 (red asterisks); N2 *vs. nhr-49* P = 0.002 (blue asterisks). **B–C: Effects of NHR-49 overexpression on lifespan of fertile worms.** **B:** N2 (black; m = 21.6±0.1, n = 75/98), *nhr-49* (purple; m = 14.4±0.2, n = 89/100, P *vs.* N2<0.0001), *NHR-49 OE* non-transgenic siblings (gray; m = 21.3±0.4, n = 83/102, P *vs.* N2 0.82), *NHR-49 OE* (red; m = 26.0±0.4, n = 47/84, P *vs.* N2<0.0001, P *vs. nhr-49*<0.0001, P *vs.* non-transgenic siblings <0.0001). **C:** N2 (black; m = 22.8±0.2, n = 81/92), *nhr-49* (purple; m = 13.7±0.1, n = 83/88, P *vs.* N2<0.0001), *nhr-49;NHR-49 OE* non-transgenic siblings (gray; m = 12.4±0.1, n = 93/98, P *vs. nhr-49* 0.02, P *vs.* N2<0.0001), *nhr-49;NHR-49 OE* (red; m = 25.1±0.3, n = 79/101, P *vs.* N2<0.0001, P *vs. nhr-49*<0.0001, P *vs.* non-transgenic siblings <0.0001). **D: Effect of NHR-49 overexpression on lifespan of** ***glp-1*** **mutants.** N2 (black; m = 21.6±0.1, n = 75/98), *glp- 1* (green; m = 31.0±0.5, n = 94/101, P *vs.* N2<0.0001), *nhr-49;glp-1* (purple; m = 14.1±0.1, n = 95/97, P *vs. glp-1*<0.0001), *glp-1;NHR-49 OE* (red; m = 35.3±0.5, n = 92/97, P *vs. glp-1* 0.001). **E, F:** **Effect of** ***daf-16*** **and** ***tcer-1*** **reduction of function on lifespan extended by NHR-49 overexpression. E:** N2 (black; m = 18.5±0.3, n = 67/109), *nhr-49* (purple; m = 12.7±0.1, n = 87/96, P *vs.* N2<0.0001), *daf-16 (*blue; 15.6±0.2, n = 56/120, P *vs.* N2 0.001), *NHR-49 OE* (green; m = 24.9±0.2, n = 40/98, P *vs.* N2<0.0001), *daf-16;NHR-49 OE* (red; m = 17.4±0.3, n = 56/96, P *vs.* N2 0.26, P *vs. daf-16* 0.26, P *vs. nhr-49*<0.0001). **F:** N2 worms grown on control vector bacteria (black; m = 20.3±0.7, n = 59/63). *NHR-49 OE* worms grown on control vector bacteria (green; m = 21.6±1.4; n = 21/71; P vs N2 on control vector 0.05), *daf-16* RNAi bacteria (red; m = 11.8±0.3; n = 39/62; P *vs.* control <0.0001) and on *tcer-1* RNAi bacteria (blue; m = 14.1±0.7; n = 40/64; P *vs.* control <0.0001). Additional control lifespans not shown in the graph: N2 worms grown on *daf-16* RNAi bacteria (m = 15.1±0.4; n = 59/63; P *vs.* N2 control <0.0001) and on *tcer-1* RNAi bacteria (m = 20.2±0.2; n = 53/65; P *vs.* N2 control 0.4). Data from additional trials is presented in S3 (B–D) and S5A (E) Tables.

Since NHR-49 expression is increased in *glp-1* mutants, we asked if elevating levels of the protein in normal animals could circumvent the need for germline removal and directly enhance longevity. We used the NHR-49::GFP animals that overexpressed the protein due to the presence of multiple extra-chromosomal arrays of the transgene (Fig. 3A). Indeed, we found that wild type, fertile worms overexpressing NHR-49 lived ∼15% longer than their non-transgenic siblings and wild-type controls (Fig. 3B, S3 Table). Intriguingly, the lifespan enhancement was greater when NHR-49 was overexpressed in an *nhr-49* mutant background. Not only was the lifespan of *nhr-49* mutants rescued to wild-type levels, it was augmented even further (Fig. 3C). These long-lived worms did not display any obvious fertility defects (S3 Figure). NHR-49 overexpression in *glp-1* mutants caused a small additional increment in their longevity as well (Fig. 3D). This lifespan increment was dependent on both *daf-16* and *tcer-1* (Fig. 3E, F and S5A Table). These data show that elevating NHR-49 levels can increase lifespan modestly without compromising fertility.

### NHR-49 controls the elevated expression of mitochondrial β-oxidation genes in germline-less animals

**Figure 4:**
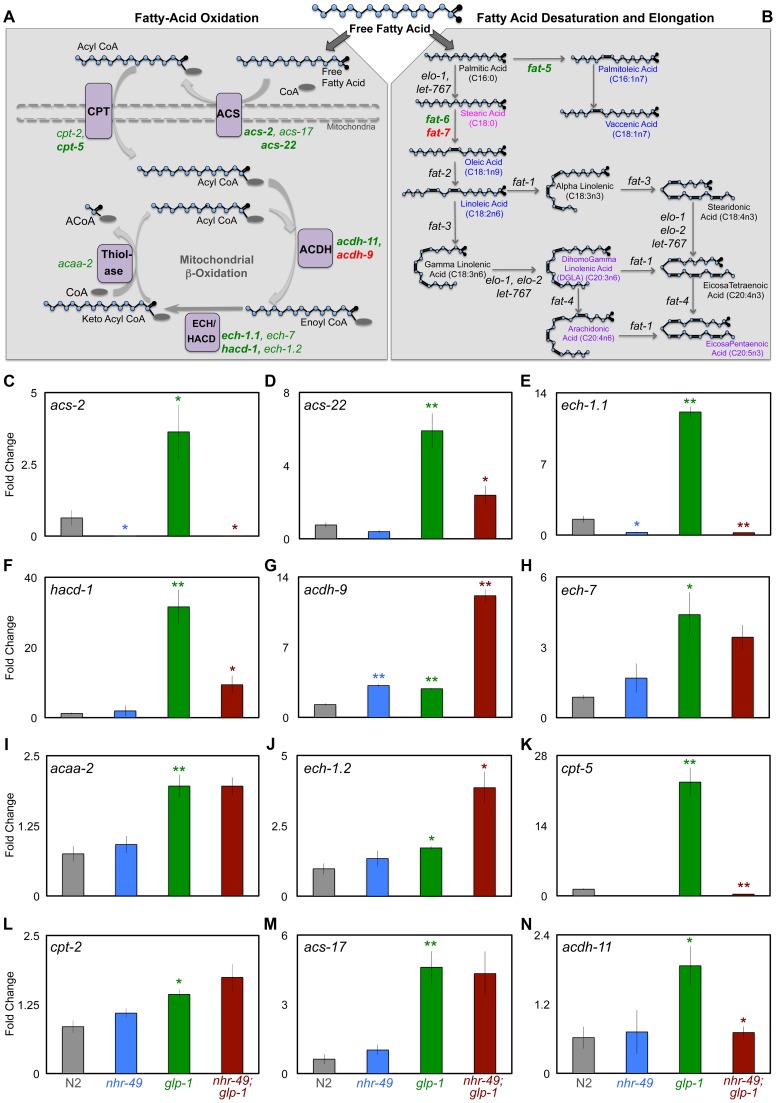
NHR-49 up-regulates the expression of genes involved in different steps of mitochondrial β-oxidation upon germline loss. **A, B: Schematic representation of the mitochondrial β-oxidation, fatty-acid desaturation and elongation pathways.** Free fatty acids can be directed for breakdown through β-oxidation in peroxisomes and mitochondria (A) or take an anabolic path through desaturation and elongation (B). **A**: Mitochondrial β-oxidation involves the repetitive action of a series of enzymes that ultimately results in the breakdown of fatty acids into acetyl CoA [43]. Enzymes that function at each of these stages are indicated in purple boxes; ACS (acyl CoA synthetase), CPT (carnitine palmitoyl transferase), ACDH (acyl CoA dehydrogenase), ECH (enoyl CoA hydratase), HACD (hydroxyl acyl CoA dehydrogenase) and thiolase. The genes encoding these enzymes that were up-regulated in *glp-1* mutants are represented in green. Of these, those dependent on *nhr-49* for their up-regulation are in bold. Genes repressed by *nhr-49* are in red. The dashed lines mark the mitochondrial membranes. **B**: The steps involved in fatty-acid desaturation and elongation that results in conversion of small SFAs to longer MUFA and PUFA species are depicted through the example of palmitic acid (C16:0) [36]. Genes up-regulated in *glp-1* mutants by *nhr-49* are highlighted in green and those repressed are in red. Fatty-acid species that showed significantly different levels between day 2 *glp-1* and *nhr-49*;*glp-1* mutants in GC/MS are represented in color: fatty acids reduced in *nhr-49*; *glp-1* mutants are in blue and those elevated are in pink. Some PUFAs were elevated only in the phospholipid fraction of *nhr-49*;*glp-1* mutants and these are highlighted in purple. For a complete list of genes involved in both these pathways see S4 Figure. **C**–**N: The expression of multiple mitochondrial β-oxidation genes is elevated in germline-depleted animals under control of** ***nhr-49*** **.** mRNA levels of mitochondrial β-oxidation genes examined by Q-PCRs performed on at least three biological replicates isolated from day 2 adults of the following strains: wild-type (N2, gray), *nhr-49* (blue), *glp-1* (green) and *nhr-49*;*glp-1* (red). All 12 genes tested showed increased expression in *glp-1* mutants. Of these, the up-regulation of 7 genes was significantly reduced or abolished in *nhr-49*;*glp-1* mutants (effect on *ech-7* did not achieve statistical significance). These included previously identified NHR-49 targets {*acs-2* (B), *cpt-5* (J), *ech-1.1* (D) and *hacd-1* (E)} as well as new ones {*acs-22* (D), *acdh-11* (N)}. *acdh-9* mRNA was elevated in *glp-1* mutants compared to N2 but further elevated in *nhr-49*;*glp-1* mutants (F). The statistical significance of the N2 *vs. glp-1*, *glp-1 vs. nhr-49*;*glp-1* and N2 *vs. nhr-49* comparisons in an unpaired, two-tailed t-test are represented by green, red and blue asterisks, respectively. Number of asterisks correspond to P values 0.05 (*), 0.005 (**) and <0.0001 (***).

During development and in response to food deprivation, NHR-49 regulates the expression of multiple genes predicted to function in mitochondrial- and peroxisomal- β-oxidation (Fig. 4A) as well as fatty-acid desaturation pathways (Fig. 4B) [36], [37]. Strikingly, along with *nhr-49*, many of these genes, were also identified as DAF-16 and TCER-1 targets in the RNA-Seq analysis mentioned above (S4 Figure; Amrit *et al.,* manuscript in preparation). This led us to ask (a) if the expression of these genes was enhanced in *glp-1* mutants, and (b) whether their up-regulation was dependent upon *nhr-49*. We focused on the mitochondrial β-oxidation genes. In Q-PCR assays, the mRNA levels of 12 genes we tested were all elevated in long-lived, *glp-1* mutants as compared to wild-type worms, although to variable degrees (Fig. 4C–N). Of these, the up-regulation of seven genes was significantly reduced or abolished in *nhr-49*;*glp-1* mutants (Fig. 4C-N). These genes encode enzymes that participate in different steps of mitochondrial β-oxidation including: i) acyl CoA synthetases (ACS; *acs-2* and *acs-22*) that catalyze the conversion of fatty-acids to acyl CoA ii) carnitine palmitoyl transferases (CPT; *cpt-2*, *cpt-5*) that transport activated acyl groups from the cytoplasm into the mitochondrial matrix and iii) acyl CoA dehydrogenases (ACDH; *acdh-9, acdh-11*), enoyl CoA hydratases (*ech-1.1, ech-7*) and thiolase (*acaa-2*) whose combined activities result in the shortening of fatty-acid moieties and generation of acetyl CoA (Fig. 4A) [43]. Thus, NHR-49 mediates the increased expression of genes involved in different steps of mitochondrial β-oxidation following germline loss. We did not observe a similar, conspicuous difference in the expression of these genes on comparing wild-type worms with *nhr-49* single mutants. The expression of two genes, *acs-2* and *ech-1.1*, was reduced in *nhr-49* mutants (Fig. 4C, E) and *acdh-9* was elevated (Fig. 4G), but the others were not significantly altered (Fig. 4 and S5 Figure). Surprisingly, worms overexpressing NHR-49 also did not exhibit a consistent change in the mRNA levels of these genes, although they are longer lived and it was conceivable that they may have elevated β-oxidation gene expression (S6 Figure). These observations suggest that germline removal may provide the impetus for a perspicuous up-regulation of β-oxidation genes by NHR-49.

To test if these gene expression changes had any relevance on the lifespan extension observed in germline-less animals, we examined the effect of RNAi knockdown of each of these genes on the longevity of *glp-1* mutants. RNAi was initiated with the onset of adulthood to circumvent developmental requirements. We found that RNAi knockdown of seven of nine genes shortened *glp-1* longevity to variable degrees (7–48%; Table 1 and S6 Table). On the other hand, RNAi knockdown of the same genes in a control strain with wild-type lifespan either had no statistically significant effect (7/9 genes tested) or an inconsistent lifespan reduction (2/9 genes) (Table 1 and S6 Table). These results underscore the singular importance of the β-oxidation genes we tested to the longevity of germline-less animals. Together, our data defined a functional role for the NHR-49-mediated up-regulation of mitochondrial β-oxidation genes in response to germline removal. Moreover, they suggested that in germline-less animals, NHR-49 triggers an increase in fatty-acid β-oxidation and this metabolic shift is critical for the consequent lifespan extension.

**Table 1:** Multiple mitochondrial fatty-acid β-oxidation genes regulated by NHR-49 are essential for the increased longevity of germline-less animals but not wild-type worms.

			RNAi Inactivation in Germline-Less Adults					RNAi Inactivation in Wild-Type* Adults			
#	Enzyme Encoded	Gene	Cosmid	n = obs/total^b^	Mean ±SD	P (vs Control^b^)	Percent Effect on Lifespan	n = obs/total^b^	Mean ±SD	P (vs Control^b^)	Percent Effect on Lifespan
		Empty Vector Control^a^			27.9±1.3			**73/73**	11.2+0.4		
		*daf-16*	R13H8.1		14.8±0.3	<0.0001	−46				
		*tcer-1*	ZK1127.9		19.0±0.6	<0.0001	−32				
		*daf-2*	Y55D5A.5					104/104	20.6+0.7	0	+84.5
1	Acyl CoA Synthetase	*acs-2*	F28F8.2	71/75	14.6±0.1	<0.01	−48	67/68	12.3±0.3	0.14	+10
2	Acyl CoA Synthetase	*acs-17*	C46F4.2	72/75	18.2±0.5	<0.0001	−35	47/47	13.3±0.3	0.01	+19
3	Acyl CoA Synthetase	*acs-22*	D1009.1	72/75	23.4±0.8	0.0001	−16	38/38	12.3±0.6	0.13	+10
4	Carnitine Palmiloyl Transferase	*cpt-2*	R07H5.2	66/66	22.8±0.8	0.0001	−18	67/71	11.7±0.3	0.55	ne
5	Acyl CoA Dehydrogenase	*acdh-11*	Y45F3A.3	60/64	23.4±1.0	0.0126	−16	101/101	11.8±0.3	0.33	ne
6	Enoyl CoA Hydratase	*ech-7*	Y105E8A.4	64/75	20.1±0.2	<0.0001	−28	41/41	12.6±0.6	0.05	+13
7	Hydroxyl Acyl CoA Dehydrogenase	*hacd-1*	R09B5.6	76/77	19.7±0.3	<0.0001	−29	77/79	10.4±0.3	0.14	- 7
8	HADHA**	*ech-1.2*	T08B2.7	66/68	24.3±1.2	0.1728	−13	26/26	11.1±0.5	0.74	ne
9	Thiolase	*acaa-2*	F53A2.7	64/71	25.9±1.0	0.0578	−7	89/92	11.8±0.4	0.22	ne

*glp-1* mutants (1A) and the sterile strain, *fer-15(b26);fem-1(hc17),* (1B) were subjected to ‘adult-only’ RNAi inactivation of mitochondrial **β-**oxidation genes whose expressions were elevated upon germline loss, many in an NHR-49-dependent manner. See methods section for experimental details and S6 Table for additional trials.

*fer-15(b26);fem-1(hc17) is a temperature sensitive strain that when grown at 25°C is sterile and used as a surrogate for wild-type, N2 in lifespan assays [69].

**ech-1.2 (T08B2.7) is orthologous to the human gene that encodes the tri-functional protein, hydroxyl acyl CoA dehydrogenase/3-ketoacyl CoA thiolase/enoyl CoA hydratase (HADHA) alpha subunit (www.wormbase.org) and a close paralog of *ech-1.1*. *ech-1.1* and *cpt-5* could not be tested due to contamination of RNAi clones. Data is shown as mean lifespan in days (Mean) ± standard error of the mean (SEM). ‘n’ refers to the number of worms observed (obs) divided by total number of worms tested in the experiment.

a some worms were censored from the analysis as described in methods.

b Empty vector control refers to worms exposed to empty vector plasmid without an RNAi insert. P values were calculated using the log rank (Mantel Cox) method.

### NHR-49 is required for maintenance of high fat levels and *de novo* lipid synthesis in germline-less animals

**Figure 5:**
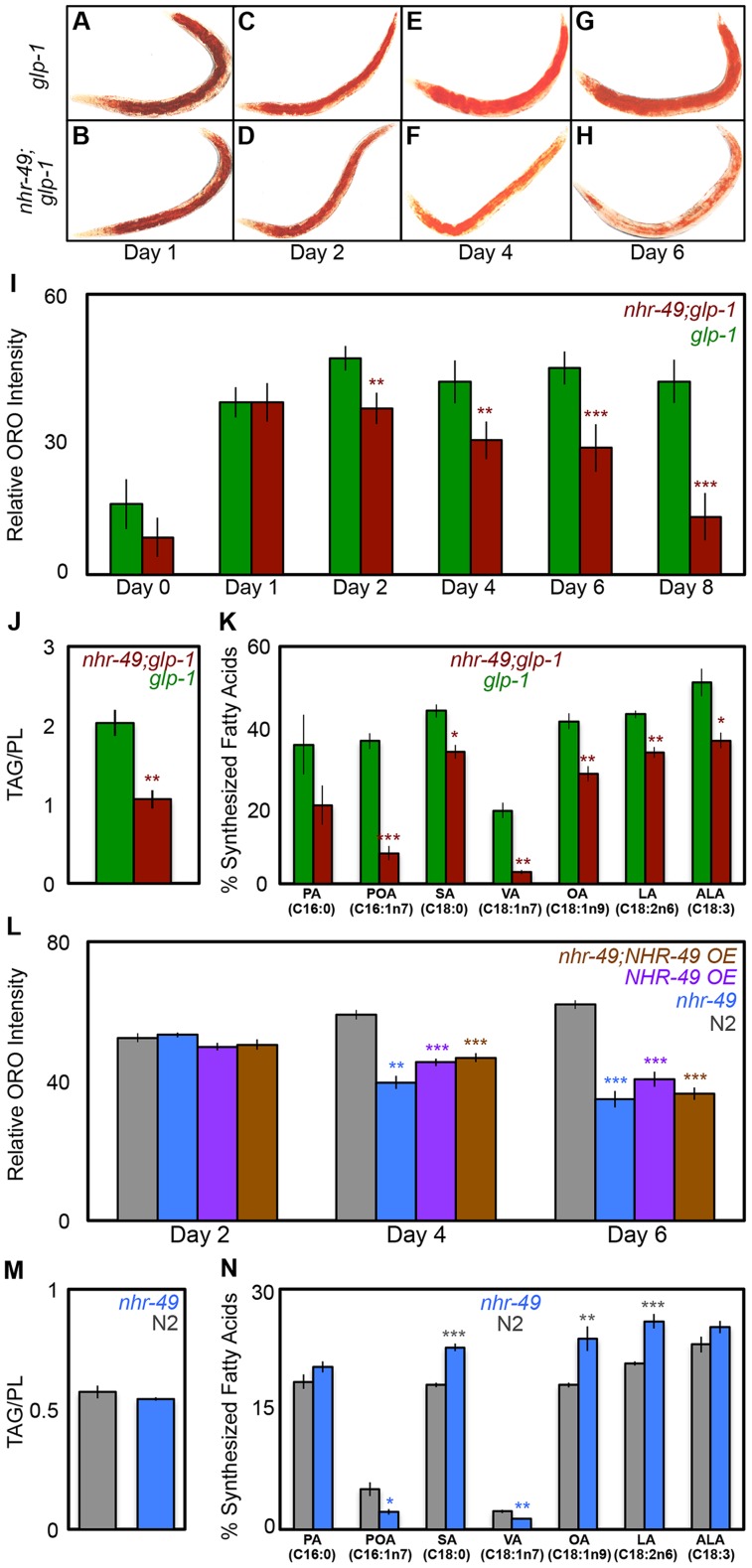
NHR-49 is required for maintenance of fat stores and *de novo* fat synthesis in germline-less adults. **A**–**I:** ***nhr-49*** **;** ***glp-1*** **mutants undergo a dramatic depletion of lipid stores during young adulthood.** Lipid levels compared between *glp-1* (A, C, E and G) and *nhr-49*; *glp-1* (B, D, F and H) through ORO staining of L4 larvae (day 0) and adults on days 1 (A, B), 2 (C, D), 4 (E, F), 6 (G, H) and 8 (I). Representative images are shown in A-H and the quantification of the data is in I. The two strains show similar fat levels on Day 1, but by day 8 the *nhr-49*;*glp-1* mutants exhibit a significant reduction in ORO staining. **J:** ***nhr-49*** **;** ***glp-1*** **mutants show decreased TAG levels.** Using GC/MS, the triglyceride: phospholipid (TAG/PL) ratio of day 2 *nhr-49*;*glp-1* adults was found to be significantly lesser than that of age-matched *glp-1* animals. **K:** ***de novo*** **fatty-acid synthesis is impaired in** ***nhr-49*** **;** ***glp-1*** **mutants.** Using a 13C isotope fatty-acid labeling assay, *de novo* fat synthesis and dietary fat absorption were compared between day 2 *glp-1* and *nhr-49*;*glp-1* adults. Individual fatty-acid species are represented on the X-axis and relative synthesis levels are on the Y-axis. Synthesis of six out of seven species was significantly reduced upon *nhr-49* reduction of function. **L:** ***nhr-49*** **mutants undergo lipid depletion with age.** Lipid levels compared between wild-type (N2, gray), *nhr-49* (blue), *NHR-49::GFP* (*NHR-49 OE,* purple) and *nhr-49;NHR-49::GFP* (*nhr-49;NHR-49 OE,* brown) strains through ORO staining of adults on days 2, 4 and 6 of adulthood. The strains show similar fat levels on day 2, but by day 6 *nhr-49* mutants as well as worms overexpressing NHR-49 display a significant reduction in ORO staining. **M:** ***nhr-49*** **mutants do not have increased TAGs.** Using GC/MS, the triglyceride: phospholipid (TAG/PL) ratio of late L4/early day 1 *nhr-49* mutants was found to be similar to that of age-matched wild-type animals. **N:** ***de novo*** **fatty-acid synthesis is disrupted in** ***nhr-49*** **mutants.** *de novo* fat synthesis and dietary fat absorption were compared between late L4/early day 1 *nhr-49* mutants and wild-type (N2) adults using the 13C isotope fatty-acid labeling assay. Synthesis of some fatty acids was reduced and that of others was increased in *nhr-49* mutants in the neutral lipid fraction (see S8 Figure for phospholipid data). A similar comparison of age-matched *glp-1* and *nhr-49*;*glp-1* adults presented a similar mixed profile with the notable exception of OA whose synthesis was reduced in *nhr-49;glp-1* mutants at both stages while in *nhr-49* mutants it was synthesized at a higher level (S8B, C Figure). By day 2, the synthesis of all fatty acids tested was uniformly reduced in *nhr-49*; *glp-1* mutants (K). All graphs were obtained by combining data from at least three independent biological replicates. Error bars indicate the standard error of the mean. Asterisks depict the statistical significance of the observed differences in an unpaired, two-tailed t-test with P values 0.05 (*), 0.005 (**) and <0.0001 (***). The color of the asterisk denotes the strain showing the observed reduction.

Germline loss results in increased triglyceride (TAG) storage in *C. elegans* [44]. Based on the observations described above, we asked if inhibiting mitochondrial β-oxidation affected the elevated fat stores of *glp-1* mutants. As a preliminary test, we compared the lipid levels of *glp-1* and *nhr-49*;*glp-1* mutants by staining the animals with the dye Oil Red O (ORO) that labels TAGs and whose estimation closely matches biochemically detected TAG levels [44]. Since β-oxidation is a lipolytic pathway, it was conceivable that *nhr-49*;*glp-1* mutants would exhibit a further increase in TAGs due to their impaired mitochondrial β-oxidation gene expression profile. However, we found that ORO levels were indistinguishable between *glp-1* and *nhr-49*;*glp-1* day 1 adults (Fig. 5A, B and I). Surprisingly, by day 2, *nhr-49*;*glp-1* adults showed a small but significant reduction in ORO staining as compared to *glp-1* mutants (Figs. 5C, D, I and S6A Figure). As the animals aged, this difference became more pronounced. By days 6–8 of adulthood, *nhr-49*;*glp-1* mutants underwent a striking loss of ORO staining (Fig. 5E–I). By comparison, *glp-1* mutants continued to show high ORO staining from day 2 till at least day 18 of adulthood (S6B Figure). To obtain a direct measure of lipid levels, we used gas chromatography/mass spectrometry (GC/MS) and found that TAG levels are indeed significantly reduced in *nhr-49*;*glp-1* mutants as compared to *glp-1* adults (Fig. 5J).

To understand why *nhr-49*;*glp-1* mutants exhibited reduced TAG levels despite diminished expression of β-oxidation genes, we explored the possibilities that (a) they consumed less food than *glp-1* mutants, and/or (b) they also experienced a simultaneous reduction in fatty-acid synthesis. We observed no difference in the pharyngeal pumping rates of the two strains, indicating that they ate similar quantities of food (S6C Figure). Next, we compared the dietary fat absorption and *de novo* fat synthesis between *glp-1* and *nhr-49*;*glp-1* mutants using a previously described 13C isotope fatty-acid labeling assay [45]. *de novo* fat synthesis was substantially reduced in *nhr-49*;*glp-1* mutants (Fig. 5K). We confirmed that this reduction was not due to repressed transcription of genes involved in initiation of fat synthesis or those mediating conversion to stored fats. mRNA levels of *pod-2* {that encodes acyl CoA carboxylase (ACC), the rate-limiting enzyme required for initiation of fat synthesis} and *fasn-1* {that encodes fatty-acid synthase (FASN-1), another key regulator of fat synthesis} were not reduced by *nhr-49* reduction of function (S7A and B Figure). Similarly, *nhr-49* did not affect the expression of *dgat-2*, a gene that encodes a rate-limiting enzyme diacylglycerol acyl transferase (DGAT) needed for conversion of diglycerides (DAGs) into TAGs (S7C Figure). Thus, our experiments showed that germline-less animals require *nhr-49* for *de novo* lipid synthesis and to retain their high TAG levels during adulthood. They suggest that impairing NHR-49 may impact other important metabolic processes besides β-oxidation such as lipid synthesis, storage and maintenance.

### *nhr-49* mutants exhibit age-related depletion of lipid stores

Since *nhr-49* mutants are short-lived compared to wild-type controls [36], [46], we asked if they also exhibited age-related fat phenotypes, and if NHR-49 played an analogous role during normal aging. *nhr-49* mutants have been reported to have higher fat. These studies predominantly relied on staining live larvae with the dye Nile Red [36], [47]–[49], an inaccurate technique for labeling fats as the dye is trafficked to the lysosome-related organelle in live animals [44], and in some cases these observation have not been corroborated by other methods [50]. Using ORO labeling, we did not observe a significant difference between wild-type, day 2 animals and age-matched *nhr-49* mutants (Fig. 5L and S6A Figure). However, while wild-type worms underwent increased fat accumulation with age, *nhr-49* mutants, similar to *nhr-49*;*glp-1*, exhibited a progressive loss of fat (Fig. 5L). Intriguingly, a similar age-related loss of ORO staining was also observed in worms overexpressing NHR-49 (Fig. 5L). These phenotypes could not be explored biochemically in the reproductively active day 2 adults of these strains due to the confounding effects of eggs and progeny (see Materials and Methods). Hence, we used late L4 larvae/early day 1 adults to compare the lipid profiles of wild-type worms and *nhr-49* mutants. GC/MS data showed that, at least in late L4/early day 1 adults, lipid levels were the same between the two strains (Fig. 5M). Since the biochemical analyses could not be extended to adults, it is formally possible that *nhr-49* mutants have elevated fat. But, our experiments strongly indicate that *nhr-49* loss of function does not increase fat accumulation. Instead, in both germline-less and normal adults, it causes an age-related loss of stored lipids. On comparing the dietary fat absorption and *de novo* lipid synthesis profiles between late L4/early day 1 *nhr-49* mutants and wild-type worms, we noticed fatty-acid specific differences. Some fatty acids were synthesized at a higher level in *nhr-49* mutants as compared to wild-type (eg., OA) whereas the synthesis of others (eg., Vaccenic Acid, C18:1n7) was reduced (Fig. 5N and S8A Figure). Together, these experiments showed that NHR-49 is required for the maintenance of TAG stores during normal aging and its absence causes *de novo* lipid synthesis abnormalities. The similarities between the phenotypes of *nhr-49* and *nhr-49;glp-1* mutants suggest a shared role for the gene in the two contexts. However, the gene expression and lifespan studies described in the previous section also point towards mechanistic and possibly functional differences in NHR-49's modulation of these processes in fertile *vs.* germline-less adults (see Discussion).

### NHR-49 enhances the desaturation of multiple fatty acids in germline-less adults

**Figure 6:**
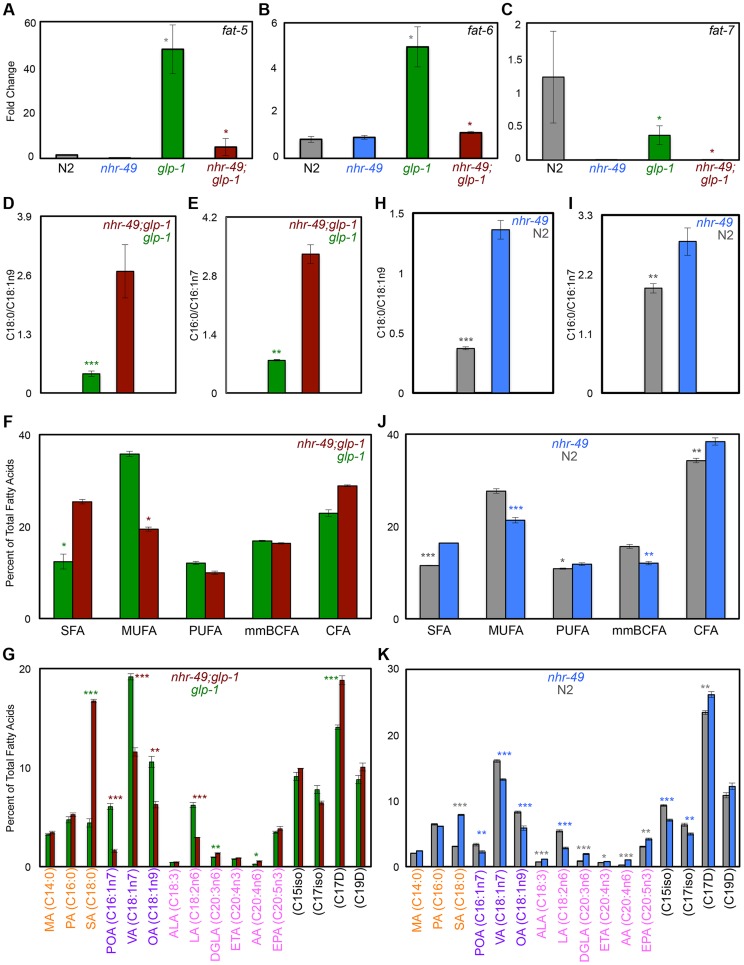
NHR-49 has a broad impact on fatty-acid desaturation in germline-less animals. **A-C: The expression of fatty-acid desaturase genes in *nhr-49*;*glp-1* mutants.** mRNA levels of *fat-5* (A), *fat-6* (B) and *fat-7* (C) were compared by Q-PCRs between day 2 adults of the following strains: wild-type (N2, gray), *nhr-49* (blue), *glp-1* (green) and *nhr-49*;*glp-1* (maroon). In accordance with previous data, *fat-5* and *fat-6* were up-regulated in *glp-1* mutants as compared to N2, whereas, *fat-7* levels were reduced [30]. In *nhr-49*;*glp-1* mutants, the expression of all three genes was diminished. **D, E: Desaturation of stearic acid (SA, C18:0) to oleic acid (OA, C18:1n9) (D) and palmitic acid (PA, C16:0) to palmitoleic acid (POA, C16:1n7) (E) in** ***nhr-49*** **;** ***glp-1*** **mutants.** The SA:OA and PA:POA ratios (Y-Axes) were obtained by analysis of neutral lipid GC/MS data of day 2 adults. SA:OA and PA:POA was significantly enhanced in *nhr-49*;*glp-1* mutants suggesting decreased desaturation resulting in the accumulation of SA and PA at the expense of OA and POA, respectively. Similar data was obtained from analysis of the phospholipid fractions (S9 Figure). **F, G: unsaturated fatty acids (UFAs) are widely reduced in** ***nhr-49*** **;** ***glp-1*** **mutants.** In both the neutral lipid (F) and phospholipid (S9 Figure) fractions of *nhr-49*;*glp-1* mutants, there was a significant reduction in the overall level of mono-unsaturated fatty acids (MUFAs) as compared to *glp-1* mutants, whereas saturated fatty acid (SFA) content was elevated. Comparisons of individual fatty acid levels in the neutral lipid pool are shown in G (see S9 Figure for phospholipid data). SFAs are labeled in orange (MA: myristic acid), MUFAs in purple (VA: vaccenic acid) and PUFAs in pink (ALA: α-linolenic acid; LA: linoleic acid; DGLA: dihomo γ-linolenic acid; ETA: eicosatetraenoic acid; AA: arachidonic acid; EPA: eicosapentaenoic acid). Black labels mono-methyl branched chain fatty acids (mmBCFA) and cyclopropane fatty acids (CFA). Y-axes indicate the percent of total fatty-acid pool contributed by each of the species indicated on the X-axes. **H-K: Comparison of neutral lipids composition of late L4/early day 1** ***nhr-49*** **mutants and age-matched wild-type worms. H, I**: SA:OA (H) and PA:POA (I) ratios are elevated in *nhr-49* mutants. **J, K**: *nhr-49* mutants show increased SFAs and reduced MUFAs. Levels of PUFAs, mmBCFAs and CFAs are altered as well. Variations in individual fatty acid levels are shown in K. Data from phospholipid fractions are shown in S10 Figure. Graphs in all panels were obtained by combining data from three independent biological replicates. Asterisks show the statistical significance of the observed differences in an unpaired, two-tailed t-test with P values 0.05 (*), 0.005 (**) and <0.0001 (***). The color of the asterisk denotes the strain showing the observed reduction.

NHR-49 regulates both fatty-acid β-oxidation and desaturation during development and nutrient deprivation [36], [37] so we asked if it impacted desaturation in the *glp-1* mutant context and/or during normal aging. The genes *fat-5, fat-6* and *fat-7* encode desaturase enzymes that catalyze the conversion of SFAs to MUFAs. FAT-5 converts palmitic acid (PA, C16:0) to palmitoleic acid (POA, C16:1n7) while FAT-6 and -7 function redundantly to convert stearic acid (SA, C18:0) to oleic acid (OA, C18:1n9) (Fig. 4B) [51], [52]. Under control of NHR-80, FAT-6/7 mediated conversion of SA to OA is necessary for the longevity of *glp-1* mutants; OA supplementation completely rescues the short lifespan of *glp-1;fat-6;fat-7* mutants to *glp-1* level [30]. We found that, similar to NHR-80, NHR-49 was also required for the changes in levels of *fat-5, fat-6* and *fat-7* observed in *glp-1* mutants (Fig. 6A–C). However, OA supplementation did not rescue the shortened lifespan of *nhr-49*;*glp-1* mutants (S7 Table) indicating other critical functions for NHR-49. On comparing the lipid profiles of *nhr-49*;*glp-1* with *glp-1* mutants through GC/MS, we observed an increased SA:OA ratio in the former, as expected (Fig. 6D and S9A Figure). In addition, the ratio of PA:POA was enhanced as well (Fig. 6E and S9B Figure). Overall, *nhr-49;glp-1* mutants exhibited a widespread decline in MUFAs and increased SFAs in both the neutral and phospholipid pools (Figs. 6F, G and S9C, D Figure). Desaturation is coupled to the elongation of fatty-acid chains that is mediated by elongase enzymes (encoded by the *‘elo’* genes in *C. elegans*). Our Q-PCR assays showed that *nhr-49* was not required for the up-regulation of *elo-1* and *elo-2* in *glp-1* mutants (S7D, E Figure) implying a selective role for the gene in desaturation. Overall, these data showed that NHR-49, similar to NHR-80, is required for SA-to-OA conversion in *glp-1* mutants. In addition, it also promotes the desaturation of other SFAs to MUFAs and PUFAs to ensure an UFA-rich lipid profile. Hence, while NHR-80 influences desaturation alone, NHR-49 modulates both desaturation and β-oxidation and has a broader effect on lipid composition. This may also explain the more severe phenotypes associated with *nhr-49* reduction-of-function.

In *nhr-49* single mutants, the levels of *fat-5* and *fat-7* mRNAs were reduced, whereas the effect on *fat-6* was inconsistent and statistically insignificant (Figs. 6A-C and S5 Figure). Despite these weak gene-expression effects, the fatty-acid profile of late L4/early day 1 *nhr-49* mutants showed increased SA:OA ratio (PA:POA ratio was increased only in neutral lipids; Figs. 6H, I and S10A, B Figure) and an increased accumulation of SFAs with a concomitant reduction in MUFAs (Figs. 6J, K and S10C, D Figure) indicating a role for NHR-49 in establishing a MUFA-rich lipid profile in normal animals too. Overall, the multiple fat phenotypes of *nhr-49*;*glp-1* mutants, the role of *nhr-49* in enhancing fatty-acid β-oxidation as well as desaturation and our biochemical and functional data together suggest that through the coordinated enhancement of β-oxidation and desaturation, NHR-49 helps establish lipid homeostasis that is critical for the survival of germline-less animals, and may also impact normal aging.

## Discussion

In this study, we show that NHR-49 promotes the longevity of germline-less adults through the increased expression of genes that mediate mitochondrial β-oxidation and fatty-acid desaturation. Our data strongly suggest that germline-removal enhances fatty-acid oxidation and desaturation through NHR-49 activity. We propose that through the synchronized up-regulation of these ostensibly disparate lipid-metabolic pathways, NHR-49 facilitates the adaptation to loss of reproductive potential (by eliminating fats designated for reproduction) and helps establish a lipid profile that favors increased lifespan (by converting SFAs into UFAs that are more conducive for cellular maintenance (S11 Figure) [53]–[56].

### Lipid homeostasis and the coupling of β-oxidation and desaturation

A key finding of our study is the identification of multiple genes predicted to function in fatty-acid β-oxidation whose expression is up-regulated following germline loss, and the strong dependence on NHR-49 for this up-regulation. These genes encode enzymes that together cover all the catalytic reactions of β-oxidation including some that are specific to the process (e.g., CPTs) [43]. While we cannot rule out the possibility that they function together in a different pathway, the simplest interpretation of our data is that these genes enhance mitochondrial β-oxidation. These data imply that germline removal causes a shift towards fatty-acid metabolism. Lipid oxidation confers several advantages over glucose metabolism such as more efficient energy production and reduced reactive oxygen species (ROS) generation [57]. In *C. elegans*, fatty-acid oxidation provides energy in other situations where stored lipids are used for long-term survival such as dauer diapause and caloric restriction [47], [58]. However, in these contexts, the animal is food deprived and not faced with the hazard of large-scale lipid accumulation due to thwarted procreation. Following germline loss, a metabolic shift towards increased β-oxidation coupled to lipid repartitioning may allow the animal to eliminate fats normally delegated for reproduction and restore lipid homeostasis, thus averting the negative consequences of loss of fertility. Such a metabolic shift can also explain the extraordinary dependence of germline-ablated animals on the presence of NHR-49, a key mediator of both oxidation and desaturation. Fatty-acid oxidation and desaturation, although independent processes, are intimately interlinked and inter-dependent. Deficiency of the mouse desaturase, SCD1, inhibits β-oxidation in cardiac cells [59]. Alternatively, impaired β-oxidation impacts lipid composition and is implicated in human dyslipidemias [60]. A coordinated up-regulation of these processes would be especially relevant for germline-less animals, since they face the dual challenges of eliminating superfluous fat and transforming their lipid profile in adaptation to an altered physiological status.

We were intrigued by the progressive depletion of stored fats, despite impaired expression of β-oxidation genes, in *nhr-49;glp1* mutants. While the precise reason for this is unknown, we postulate that it may be due to the simultaneous inhibition of β-oxidation and desaturation that causes accumulation of free fatty acids (FFAs) [61]. FFAs stimulate insulin release and serve as key signaling molecules. But their chronic accrual causes deregulated insulin secretion and apoptosis in pancreatic β cells [62] and insulin resistance in muscle and liver cells [63]. Impaired fatty-acid oxidation and non-metabolized SFAs are implicated as the primary agents underlying lipotoxicity [64]. We observed a significant increase in such SFAs in *nhr-49*;*glp-1* mutants. Hence, it is conceivable that in *nhr-49*;*glp-1* mutants inadequate mobilization of fat stores and impaired desaturation together cause FFA accretion and an energy imbalance that may lead to early death. Further studies will be needed to test this hypothesis and unravel the molecular basis of this intriguing phenotype.

The requirement of NHR-49 for enhancement of both β-oxidation and desaturation following germline removal distinguishes the protein from other regulators such as NHR-80 which influences desaturation, especially SA to OA conversion. In our experiments, NHR-49 also had a wider impact on the fatty-acid composition of *glp-1* mutants. Besides SA, *nhr-49*;*glp-1* mutants exhibited reduced desaturation of multiple fatty acids including PA conversion to POA. They displayed overall reduction in MUFAs and PUFAs and a concomitant increase in SFAs (Fig. 6). These data suggest a broader role for NHR-49 in the increased fatty-acid desaturation associated with germline-loss.

### The regulation of NHR-49 by DAF-16 and TCER-1

Two independent approaches led us to the identification of DAF-16 and TCER-1 as regulators of *nhr-49*: the RNAi screen described here and an RNA-Seq study designed to identify DAF-16 and TCER-1 targets (Amrit *et al.,* manuscript in preparation). NHR-49::GFP confirmed the RNA-Seq and Q-PCR data. Loss of *daf-16* almost completely abolished NHR-49::GFP in *glp-1* mutants but had no impact in fertile adults (Fig. 2). TCER-1 also specifically enhanced NHR-49 in a *glp-1* background. These observations provide clues as to how reproductive stimuli may modulate somatic metabolism. Since germline loss triggers intestinal nuclear relocation of DAF-16 and elevated TCER-1 expression [20], [23], it is possible that these two events stimulate increased *nhr-49* transcription. But, it is not clear at present if *nhr-49* is a direct DAF-16 target because we did not find a canonical DAF-16-Binding Element (DBE) [65] in the promoter used in our study. The strong DAF-16-dependence in *glp-1* mutants also distinguished NHR-49 from NHR-80 whose up-regulation in germline-ablated animals is largely DAF-16 independent [30]. While the lifespan of *daf-16*;*glp-1* mutants is increased by NHR-80 [30], NHR-49 overexpression in these animals did not rescue longevity significantly (S5B Table).

### The role of NHR-49 during normal aging

The short lifespan of *nhr-49* mutants led us to explore its role during normal aging. *nhr-49* loss causes similar age-related fat loss and biochemical deficits in both germline-less and wild-type adults, but we also noticed mechanistic and regulatory differences between the two paradigms. For instance, *nhr-49;glp-1* mutants exhibited a consistent reduction in the *de novo* synthesis of OA, an important determinant of *glp-1* longevity [30], whereas, *nhr-49* mutants appeared to synthesize more of it, at least at late L4/early day 1 stage. Similarly, DAF-16 and TCER-1 mediated increased NHR-49 expression in *glp-1* mutants but were not needed for the basal expression in wild-type adults. Further, NHR-49 was required for the up-regulation of multiple mitochondrial β-oxidation genes in *glp-1* mutants, whereas, their levels were largely unchanged by its depletion in fertile adults. RNAi knockdown of these genes also impacted *glp-1* longevity selectively (Table 1). These data suggest that germline-less animals experience enhanced β-oxidation and are more dependent upon it for survival, whereas basal levels are maintained in young, fertile adults. In the light of these differences, the similarities in age-related fat loss and fatty-acid composition defects between *nhr-49* and *nhr-49*;*glp-1* mutants are intriguing. One possible explanation for these contradictory observations is that *nhr-49* controls the same pathway in the two situations but through the regulation of different targets, a premise supported by the considerable redundancy observed in *C. elegans* mitochondrial β-oxidation genes. It is also plausible that NHR-49 influences a different process in fertile adults whose inactivation also leads to progressive fat loss. Interestingly, other longevity-promoting genes exhibit similar phenotypes. For instance, HSF-1 is needed for the longevity of *daf-2* mutants and their enhanced stress resistance. But, its depletion also shortens lifespan and increases stress-susceptibility in wild-type worms [26]. Similarly, DAF-16 and SKN-1 are both essential for *daf-2* longevity and stress-resistance and they are also critical for normal worms’ ability to mount a response against oxidative stress, pathogen attack and other noxious stimuli [66]–[68]. Mutations in both genes shorten lifespan in wild-type worms [66], [67], though not to the extent seen in *hsf-1* and *nhr-49* mutants. These similarities may reflect a common mechanism by which normal cellular and metabolic pathways are leveraged and enhanced by an organism to cope with major physiological changes, and how this may in turn lead to a change in the length of life. Our results suggest that increased mitochondrial β-oxidation and transformation of the lipid profile into one enriched in UFAs may not only allow adaptation to germline loss but also be beneficial to normal aging animals.

## Materials and Methods

### Worm culture and lifespan analysis

All strains were maintained by standard techniques at 20°C. Lifespan experiments were conducted as described previously and have been discussed in detail elsewhere [69]. For all lifespan assays that involved the *glp-1* genetic background, eggs were incubated at 20°C for 2–6 h, transferred to 25°C to eliminate germ cells, then shifted back to 20°C on day 1 of adulthood (∼72 h later) for the rest of their lifespan. For *fer-15;fem-1* lifespans, eggs were similarly transferred to 25°C to induce sterility and left at the same temperature for life. For lifespans with *daf-2* mutants, worms were grown at 15°C till L4 stage and then transferred to 20°C for life. All other lifespan assays were performed at 20°C. In all cases, the L4 stage was counted as day 0 of adulthood. Fertile strains were transferred every other day to fresh plates until progeny production ceased. For lifespans performed with transgenic strains, eggs were transferred to fresh plates and after 48 h scored under a Leica M165FC microscope with a fluorescence attachment (Leica Microsystems, Wetzlar, Germany). Transgene-carrying, fluorescent L4 larvae (day 0) were separated from their age-matched, non-transgenic siblings. The latter were used as internal controls in the same experiment. For whole-life RNAi experiments, worms were exposed to RNAi clones from hatching by transferring eggs to RNAi plates. For adult-only RNAi lifespans, the worms were grown on *E. coli* OP50 till day 0 and then transferred to freshly-seeded RNAi plates for the rest of adulthood. pAD12, an empty vector plasmid without an RNAi insert [42] was used as the control in all RNAi lifespans along with pAD43 [42] and *tcer-1* RNAi constructs to knock-down *daf-16* and *tcer-1*, respectively. Data from animals that crawled off the plate, exploded, bagged, or became contaminated were censored on the day the abnormality was observed. Stata 10.0, 8.2 (Stata Corporation, Texas, USA) and OASIS (Online Application of Survival Analysis, http://sbi.postech.ac.kr/oasis) were used for statistical analysis. P-values were calculated using the log-rank (Mantel–Cox method) test. The complete genotypes and pertinent details of all the strains used in this study are given in S8 Table.

### NHR RNAi ‘sub-library’ creation and screen

According to Wormbase WS239, 283 genes are annotated as nuclear hormone receptors (www.wormbase.org). Of these, we could isolate 429 clones targeting 259 *nhr* genes from the feeding RNAi feeding libraries created by the laboratories of Julie Ahringer and Marc Vidal [39], [40]. This ‘sub-library’ was screened to identify RNAi clones that suppressed the up-regulation of *Pstdh-1/dod-8::gfp* in a long-lived *glp-1* mutant using the strain CF2573 [23]. Briefly, RNAi clones were inoculated overnight at 37°C in LB medium containing 100 µg/ml ampicillin. 100 µl culture of each clone was seeded onto NGM plates with ampicillin (100 µg/ml) and supplemented with 1 mM IPTG. Synchronized eggs of CF2573 were isolated by hypochlorite treatment and seeded onto freshly-seeded RNAi plates. After 4–6 h at 20°C the plates were moved to 25°C for ∼70–72 h and then screened under a Leica M165FC microscope with a fluorescence attachment (Leica Microsystems, Wetzlar, Germany). In addition to pAD12, multiple random clones were also used as baseline negative controls (since pAD12 causes a modest, non-specific reduction in fluorescence in all GFP-expressing strains). All screen plates were independently examined by two observers. Clones identified by both observers were tested in three subsequent trials (S1 Table). All confirmed suppressor (and some enhancer) RNAi clones were confirmed by sequencing (M13-forward primer) and before any experiment, RNAi clones were tested by PCR (T7 primers).

### NHR-49:: GFP construction

To generate the *Pnhr-49::nhr-49::gfp* construct, 6.6 kb region of *nhr-49* gene (4.4 kb comprising the coding region covering all *nhr-49* transcripts and 2.2 kb sequence upstream of the first *nhr-49* exon) was amplified with primers modified to introduce PstI and SalI restriction sites (forward 5′ gctagCTGCAGgaccagaaagagcaagagccaatattct 3′; reverse 5′ taagcaCCCGGGtcgagcatatgattattctgctcactg 3″). The amplified product was cloned into the GFP expression vector pPD95.77 (Addgene plasmid 1495). The full-length *nhr-49* fragment was inserted upstream of, and in frame with, GFP at the PstI and SalI sites (pAG4). To generate the NHR-49::GFP expressing worms, *Pnhr-49::nhr-49::gfp* (pAG4) was injected at a concentration of 25 ng/µL or 100 ng/µL with 3.75 ng/uL or 15 ng/µL of *Pmyo-2::mCherry* co-injection marker, respectively. Three to six independent stable transgenic lines were generated for each of the genetic backgrounds in which the transgene was injected. Transgenic strains were maintained by picking fluorescent worms in each generation. The strains generated for this study are listed in S8 Table.

### GFP assays

For GFP assays involving NHR-49::GFP, eggs were transferred to freshly-seeded *E. coli* OP50 or RNAi plates, incubated at 20°C for 2–6 h, transferred to 25°C (to eliminate germ cells in strains containing *glp-1* mutation), then shifted back to 20°C on day 1 of adulthood. GFP assays were conducted on day 2 of adulthood, using the Leica MZ16F stereomicroscope. All assays were performed blind after initial familiarization with GFP levels in control plates by the experimenter.

### Imaging

For imaging purposes, 6 to 10 worms were immobilized in 35 mm optical glass bottomed dishes (MatTek Corporation, Ashland, MA) with 6 µl of 0.1 mM sodium azide in PBS. Confocal images were taken using a Leica TCS SP8 microscope. GFP fluorescence was illuminated using a 488 nm argon laser line with a 63×1.4NA oil Apochromat CS2 objective. Fluorescence was captured using a spectral HyD detector over ∼100 Z-planes. Confocal images were visualized, rendered and analyzed using Volocity Visualization Software (v 5.4, PerkinElmer).

### ORO staining

ORO staining was done as described in earlier [44]. Briefly, 0.5 g ORO (Sigma-Aldrich St. Louis, MO) was dissolved in 100 mL isopropanol and the solution was equilibrated for four days. One day before staining, the stock solution was diluted to 60% with water and filtered twice on the day of the experiment through a 0.22 µm filter. 30–40 adults were picked into a 1.5 mL tube containing 1×PBS, washed twice with 1×PBS pH 7.4 and settled by spinning at 2000 rpm for 1 min. The worms were then re-suspended in 120 µL PBS to which an equal volume of 2×MRWB buffer was added. Samples were rocked gently for 1 h at room temperature and centrifuged at 2000 rpm for 1 min. The buffer was aspirated, worms washed with PBS, re-suspended in 60% isopropanol and incubated for 15 minutes at room temperature. After 15 minutes, the 60% isopropanol solution was removed and worms were then incubated overnight with rocking in 1 ml filtered 60% ORO stain. Next day the dye was removed after allowing worms to settle, and 200 µL of 1×PBS 0.01% Triton X-100 was added. Animals were mounted and imaged with using a Leica M165FC microscope mounted with a Retiga 2000R camera (Q Imaging, Burnaby, British Columbia, Canada). Images were captured with the QCapture Pro7 software (Q Imaging, Burnaby, British Columbia, Canada) and quantified using ImageJ software (NIH).

### Q-PCRs

To perform Q-PCRs, total RNA was isolated using mirVana miRNA Isolation Kit (Ambion, Austin, TX) from approximately 5,000 day 2 worms of each strain grown under identical conditions. RNA was treated with DNase I, (Sigma-Aldrich St. Louis, MO) and cDNA was prepared from 1 µg of total RNA in a 20 µL reaction using a ProtoScript first strand cDNA synthesis kit (New England Biolabs, Beverly, MA, USA). For comparing mRNA levels from strains carrying extra-chromosomal transgenes, fluorescent worms were picked on to a 10 cm NGM plate seeded with *E. coli* OP50 and allowed to lay eggs that were maintained at 20°C. On day 1 of adulthood, worms were washed with M9 and transferred to a NGM plates seeded with *E. coli* OP50 to prevent starvation. Transgenic worms were isolated on day 2 using a Leica MZ16F stereomicroscope (Leica Microsystems, Wetzlar, Germany) with standard fluorescence filters. For each strain, approximately 200 worms were used for RNA isolation. Total RNA was extracted with TRIzol (Ambion, Austin, TX) and converted to cDNA as described above. Q-PCRs were performed using an ABI 7000 machine (Applied Biosystems). PCR reactions were undertaken in 96-well optical reaction plates (ABI PRISM N8010560). A 25 µL PCR reaction was set up in each well with 12.5 µL SensiMix SYBR Hi-ROX Kit (Bioline, USA), 1/20^th^ of the converted cDNA and 0.25 M primers. For every gene at least three independent biological samples were tested, each with three technical replicates. Primers used in this study are listed in S9 Table.

### Pharyngeal pumping assay

The pharyngeal pumping assay was done as previously described [70]. Briefly, age matched *glp-1* and *nhr-49;glp-1* worms were obtained by picking eggs on to *E. coli* OP50 seeded plates. On the day of counting one worm was transferred to a freshly seeded *E. coli* OP50 plate and allowed to recover for 2–5 minutes. Pumping rate was determined by counting the contraction of the terminal bulb of the pharynx for 30 seconds under a dissecting microscope. The counting was repeated 4 more times to get the average. After the fifth replicate, the worm was moved to a freshly seeded *E. coli* OP50 plate. Pumping rate was measured on day 2, 4 and 6 of adulthood.

### Reproductive health assays

To assess reproductive health, brood size, percentage of hatching and oocyte ratio were calculated, using at least 10 worms per strain, per biological replicate, as described previously [70].

### Stable isotope labeling and lipid purification

For each strain, gravid adults were bleached to obtain approximately 15,000 eggs that were transferred to NGM plates seeded with *E. coli OP50* for growth. The plates were incubated for 2 hours at 20°C and then transferred to 25°C for growth to desired stage. For experiments performed with day 2 sterile adults, the plates were transferred back to 20°C after 72 hours for another 18 hours of additional growth and then transferred to prepared stable isotope plates for 6 hours. The same protocol was followed harvesting worms at late L4/early day 1 adults, except for harvesting after 52 hours (N2 and *glp-1*) or 64 hours (*nhr-49* and *nhr-49;glp-1*). The additional growth time was provided for *nhr-49* mutant strains to compensate for their developmental delay under large-scale growth conditions. By day 2 of adulthood, the altered *de novo* synthesis and fatty-acid composition profiles of *nhr-49;glp-1* mutants were similarly changed when compared to *nhr-49;glp-1* mutants harvested together with *glp-1* (simultaneous 96 hour harvest) or after a 12 hour delay (108 hour harvest) (S12 Figure). Larvae and adult animals utilize their fatty acids differently. In growing larvae, large quantities of lipids are used to build membranes and establish lipid stores, whereas in fully-grown adults, they are utilized to fulfill the demands of reproduction. Using wild-type N2 and other fertile strains in lipid assays confounds the results as we begin to see the metabolic profile of the progeny skew the data as early as day 2 (Shaw Wen-Chen and Carissa Olsen, unpublished data). To circumvent this, we used late L4 larvae/early day 1 adults for our lipidomic studies with fertile strains.

The stable isotope plates were prepared as previously described; in short, each plate was seeded with a 1∶1 ratio of ^12^C-bacteria and ^13^C-bacteria grown respectively in LB or Isogro media (98.5% ^13^C-enriched media, Sigma-Aldrich). The animals were harvested, washed in M9 three times, and frozen in a dry ice/ethanol bath before being stored at −80°C until processed. Total lipids were extracted as previously described with the following modifications [45]. Briefly, standards were added to each sample (1,2-diundecanoyl-sn-glycero-3-phosphocholine, Avanti Polar Lipids, for PLs and tritridecanoin, Nu-Chek Prep, for TAGs) before the start of the extraction procedure. The lipids were extracted with 2∶1 chloroform:methanol for 90 minutes at room temperature while shaking continuously. Residual carcasses were pelleted by centrifugation and the extracted lipids were transferred to fresh tubes and dried under a constant nitrogen stream. Dried lipids were re-suspended in 1 mL chloroform and loaded onto a pre-equilibrated solid phase exchange (SPE) columns (100 mg capacity, Fisher Scientific). Lipid classes were eluted from the column in the following order: neutral lipids in 3 ml of chloroform, glycosphingolipids in 5 ml of acetone:methanol (9∶1) and then phospholipids in 3 ml of methanol. Purified lipids were dried under nitrogen, re-suspended in methanol/2.5% H_2_SO_4_ and incubated for 1 h at 80°C to create fatty acid methyl esters (FAMEs) that were analyzed by gas chromatography/mass spectrometry (GC/MS) (Agilent 5975GC, 6920MS).

### Determination of fatty acid composition and *de novo* fatty acid synthesis

The relative abundance of fatty acids in each class was determined for all the major fatty acid species in the nematode as previously described [45]. To quantify TAG and PL yields, total PL and TAG abundance were normalized using the added standards, and data were presented as a TAG:PL ratio. *de novo* synthesis was calculated through a series of described equations which allow for the quantification of the amount of each fatty acid species generated from synthesis by determining the abundance of each isotopomer [45]. The synthesis numbers reported here represent the amount of ^13^C-labeled fatty acids derived from synthesis when compared to the total amount of fatty acids newly incorporated into the animal.

## Supporting Information

Figure S1 The expression of NHR-49::GFP at different developmental stages (A) and upon *nhr-49* RNAi (B, C). A: Representative image of a group of NHR-49::GFP animals in different developmental stages. GFP is widely expressed in eggs, and L1-L4 larvae (L1, L3, L4 shown here). B, C: Young adults of NHR-49::GFP grown on bacteria expressing control (empty) vector (B) and bacteria expressing *nhr-49* dsRNA (C). GFP expression is reduced in all somatic cells except neurons.
(TIF)

Figure S2 Effect of DAF-16 and TCER-1 on *nhr-49* expression in normal adults. Relative mRNA levels of *nhr-49* measured by Q-PCR in wild-type, N2 worms (wt, gray), *nhr-49* (blue), *daf-16* (red) and *tcer-1* (orange) mutants on second, fourth and sixth days of adulthood. *nhr-49* mutants have significantly reduced expression, but no change is observed in *daf-16* or *tcer-1* mutants. mRNA levels rise with age in all the strains. Statistical significances of the observed differences between different strains and ages in unpaired, two-tailed t-tests are shown in the tables. Data shown is obtained from at least three independent biological replicates.
(TIF)

Figure S3 NHR-49 overexpression does not impair the reproductive health of normal, fertile animals. **A-C:** Bar graphs represent the comparisons of reproductive health measures between wild type (N2, gray), *nhr-49* mutants (blue), NHR-49::GFP overexpressing worms (green) and the non-transgenic, control siblings of NHR-49::GFP worms (olive). **A: Brood Size:** total number of eggs laid during the lifetime of an animal. **B: Viability:** fraction of eggs laid that successfully hatch and develop into adults. **C: Premature oocyte production:** Older hermaphrodites and those with impaired fertility lay down unfertilized oocytes. The ratio of number of oocytes laid to the number of eggs laid (oocyte ratio) gives a measure of the fecundity of the animal. NHR-49 overexpression does not impact any of these measures negatively. Data shown here is combined from three independent biological replicates, in each of which at least ten adults were examined. Error bars display standard error of the mean, and the tables under each panel depict the statistical significance of the observed differences in an unpaired, two-tailed t-test.
(TIF)

Figure S4 Multiple genes involved in fatty-acid oxidation, desaturation and elongation are regulated by NHR-49 and overlap with DAF-16 and TCER-1 targets in germline-less animals (modified from Amrit *et al.,* manuscript in preparation). The genes predicted to function in different steps of peroxisomal and mitochondrial β-oxidation (top and middle panels) and fatty-acid desaturation and elongation (bottom panel) are depicted in individual rectangles. Cosmid numbers are provided in brackets next to each gene. Genes identified previously as NHR-49 targets [36], [37], [46] are highlighted with purple rectangles. They show a significant overlap with genes identified as DAF-16 and TCER-1 targets in an RNA-Seq study (Amrit *et al.,* manuscript in preparation) represented here as colored rectangles: DAF-16 targets (yellow), TCER-1 targets (cream) and joint targets (blue). Genes up-regulated by these proteins are shown in green font and those repressed are in red font. The enzymes produced by β-oxidation genes are depicted under each category. ACS: acyl CoA synthetase; CPT: carnitine palmitoyl transferase; ACDH: acyl CoA dehydrogenase; ECH: enoyl CoA hydratase; HACD: hydroxyl acyl CoA dehydrogenase. Free fatty acids are broken down to acetyl CoA moieties by β-oxidation. They can also undergo desaturation and elongation to give rise to larger, unsaturated species that can be stored as triglycerides or incorporated into membranes. Genes involved in the poly-unsaturated fatty acid (PUFA) synthesis (i) and branched chain fatty acid synthesis (ii) pathways are shown here. In addition, lipid binding proteins (LBP) and fatty acid binding proteins (FABP) that transport fatty acids and are important for these processes are also included.
(TIF)

Figure S5 mRNA levels of β-oxidation and desaturation genes up-regulated in *glp-1* mutants in an NHR-49-dependent manner examined in *nhr-49* mutants and NHR-49::GFP strains. Relative mRNA levels measured by Q-PCR in day 2 adults of wild-type, N2 worms (wt, gray), *nhr-49* mutants (blue), *NHR-49::GFP* (*NHR-49 OE*, maroon) and *nhr-49;NHR-49::GFP* (*nhr-49;NHR-49 OE*, red) strains. *nhr-49* level was reduced in *nhr-49* mutants, and elevated in the NHR-49 OE strains, as expected. mRNA levels of the target genes were not elevated. Statistical significances of the observed differences between different strains in unpaired, two-tailed t-tests are shown in the table. Data shown is obtained from at least three independent biological replicates.
(TIF)

Figure S6 *glp-1* mutants exhibit high fat levels for significant fraction of adulthood. **A:** Bar graphs represent the quantification of fat levels estimated based on the intensity of ORO staining in day-2 wild type (N2), *nhr-49* (blue), *glp-1* (green) and *nhr-49;glp-1* (red) animals. Both *glp-1* and *glp-1;nhr-49* mutants show significant increase in staining compared to N2 and *nhr-49*. *nhr-49*;*glp-1* fat levels are modestly but significantly lesser than *glp-1* adults. Error bars display standard error of the mean, and asterisks depict the statistical significance of the observed differences in an unpaired, two-tailed t-test with P<0.0001 (***). Data shown here is obtained from 3 independent biological replicates in which all four strains were tested **simultaneously**. **B: Representative image of ORO staining in day 18** ***glp-1*** **adult.** *glp-1* mutants continued to show high fat accumulation in intestinal cells on day 18 and even up to day 30 (not shown) of adulthood (by comparison, *nhr-49*; *glp-1* mutants lost almost all intestinal fat by day 8 and the entire population perished by day 15). **C:** ***nhr-49*** **;** ***glp-1*** **mutants exhibit normal rate of pharyngeal pumping.** Number of pharyngeal pumps per minute (Y-axis) were compared between *glp-1* and *nhr-49*;*glp-1* adults on days 1, 2, 4 and 6 (X-axis). No significant difference was observed at any time point.
(TIF)

Figure S7 The effect of *nhr-49* mutation on expression genes involved in initiation of fatty-acid synthesis and elongation. **A–C:** Relative mRNA levels measured by Q-PCR of key genes involved in initiation of fat synthesis, *pod-2* (A) and *fasn-1 (*B), compared between day 2 wild-type worms (wt, gray), *nhr-49* (blue), *glp-1* (green) and *nhr-49;glp-1* (maroon) mutants. In C, the expression of *dgat-2* that encodes a diacylglycerol acyl transferase (DGAT) enzyme is probed. DGAT-2 is a rate-limiting enzyme needed for diglyceride (DAG) to triglyceride (TAG) conversion. **D, E:** Relative mRNA expression of elongase-encoding genes, *elo-1* and *elo-2,* compared by Q-PCR between the same strains. In all panels, the X-axis shows the strains and Y-axis the fold change in expression. Error bars display standard error of the mean and asterisks depict the statistical significance of the observed differences in an unpaired, two-tailed t-test with P values 0.05 (*) and 0.005 (**). Data shown is obtained from at least three independent biological replicates.
(TIF)

Figure S8 *de novo* fatty-acid synthesis is impaired in late L4/early day 1 adults carrying the *nhr-49* mutation. Using a 13C isotope fatty-acid labeling assay, *de novo* fat synthesis and dietary fat absorption were compared between late L4/early day adults of wild-type, N2 (wt) worms and *nhr-49* single mutants (A) and age-matched *glp-1* and *nhr-49*;*glp-1* mutants (B, C). Neutral lipid data is shown in solid bars (B) and phospholipid data in striped bars (A, C). Individual fatty-acid species are represented on the X-axis and relative synthesis levels are on the Y-axis. Graphs in all panels were obtained by combining data from three independent biological replicates. Asterisks show the statistical significance of the observed differences in an unpaired, two-tailed t-test with P values 0.05 (*), 0.005 (**) and <0.0001 (***). The color of the asterisk denotes the strain showing the observed reduction.
(TIF)

Figure S9 Effect of *nhr-49* mutation on fatty-acid composition in germline-less animals. Comparisons of fatty-acid profiles between *glp-1* (green) and *nhr-49;glp-1* (maroon) mutants' phospholipids (PL, striped bars) isolated from day 2 adults (A–D) and neutral lipids (NL, solid bars) as well as phospholipids isolated from L4/early day 1 adults (E–L). **A, E, G:** SA:OA ratio is increased in *nhr-49;glp-1* mutants in both lipid populations and at both ages. **B, F, H:** PA:POA ratio is increased in *nhr-49;glp-1* mutants at both ages in the neutral lipid population (B, F), but not in the phospholipids of late L4/early day 1 adults (H). **C, D, I–L: Overall MUFA levels are reduced and SFAs elevated in** ***nhr-49*** **;** ***glp-1*** **mutants.** In the phospholipid fraction of day 2 adults (C, D) and in both the neutral and and phospholipid fractions of late L4/early day 1 adults (I–L), *nhr-49*;*glp-1* mutants showed a significant reduction in the overall level of MUFAs, whereas SFA content was elevated. Similar overall profiles were observed at both stages with some exceptions. PUFA levels were significantly increased in the phospholipids of *nhr-49;glp-1* mutants at both ages, whereas they were reduced in the neutral lipid fractions at both ages (the effect did not achieve statistical significance for day 2 adults, Fig. 6H). Comparisons of individual fatty acid levels are shown in D, K and L. In K and L, SFAs are labeled in orange (MA: myristic acid), MUFAs in purple (VA: vaccenic acid) and PUFAs in pink (ALA: α-linolenic acid; LA: linoleic acid; DGLA: dihomo γ-linolenic acid; ETA: eicosatetraenoic acid; AA: arachidonic acid; EPA: eicosapentaenoic acid). Black labels mono-methyl branched chain fatty acids (mmBCFA) and cyclopropane fatty acids (CFA). Y-axes indicate the percent of total fatty-acid pool contributed by each of the species indicated on the X-axes. Asterisks show the statistical significance of the observed differences in an unpaired, two-tailed t-test with P values 0.05 (*), 0.005 (**) and <0.0001 (***). The color of the asterisk denotes the strain showing the observed reduction.
(TIF)

Figure S10 Comparison of fatty-acid composition of phospholipids isolated from late L4/early day 1 *nhr-49* mutants and age-matched wild-type worms. **A, B:** SA:OA ratio is elevated (A) but not PA:POA ratio (B) in *nhr-49* mutants. **C, D:** *nhr-49* mutants show increased overall SFAs and reduced MUFAs. Levels of PUFAs and mmBCFAs are altered as well (C). Variations in individual fatty acid levels are shown in D. Graphs in all panels were obtained by combining data from three independent biological replicates. Asterisks show the statistical significance of the observed differences in an unpaired, two-tailed t-test with P values 0.05 (*), 0.005 (**) and <0.0001 (***).The color of the asterisk denotes the strain showing the observed reduction.
(TIF)

Figure S11 Schematic representation of the proposed model to explain NHR-49 function in promoting the longevity of germline-less animals. Following germline loss, NHR-49 is up-regulated by the joint activity of DAF-16 and TCER-1. NHR-49, in turn, mediates the up-regulation of genes involved in fatty-acid β-oxidation and desaturation. The synchronized enhancement of these processes allows the animal to adapt to loss of fertility and orchestrate a lipid-homeostasis profile that supports longevity. Red arrows indicate the steps for which evidence is provided in this study.
(TIF)

Figure S12 de novo lipid synthesis and fatty-acid comparison between day 2 glp-1 adults and nhr-49;glp-1 mutants harvested after a – hour delay. **A, B:** *de novo* fat synthesis and dietary fat absorption data obtained through 13C isotope fatty-acid labeling assay compared between *glp-1* (green) and *nhr-49*;*glp-1* (maroon) (B, C). Neutral lipid data is shown in solid bars (A) and phospholipid data in striped bars (B). Individual fatty-acid species are represented on the X-axis and relative synthesis levels are on the Y-axis. **C, D:** Comparisons of fatty-acid profiles between the same strains through GC/MS analysis. Please note that the order of representation of individual fatty acids in C and D is different from that used in other graphs in the article.
(TIF)

Table S1 Retests of select ‘*nhr’* RNAi clones found to suppress the up-regulation of GFP in *glp-1*;*Pstdh-1::GFP* worms in RNAi screen. RNAi clones that were found to impair GFP expression in *glp-1*;*Pstdh-1/dod-8::gfp* day 2 adults were retested thrice by one (retest #3) or two independent observers. The results are tabulated along with the identities of the genes and their respective cosmid numbers. MV refers to an RNAi clone obtained from the Vidal collection [39]. All other clones were from the Ahringer library [40].
(PDF)

Table S2 Effect of RNAi inactivation of select ‘*nhr’* RNAi clones on longevity of *glp-1* mutants. *glp-1* mutants were subjected to RNAi inactivation of ‘*nhr’* genes found to be required for the up-regulation of *Pstdh-1/dod-8::gfp* following germline loss. See methods section for experimental details. Data from four independent trials are shown here, represented as mean lifespan in days (Mean) ± standard error of the mean (SEM). ‘n’ refers to the number of worms observed (obs) divided by total number of worms tested in the experiment. ^a^ some worms were censored from the analysis as described in methods. ^b^ Control (Ctrl) refers to worms exposed to control empty vector plasmid without an RNAi insert. P values were calculated using the log rank (Mantel Cox) method.
(PDF)

Table S3 Effect of NHR-49 loss-of-function and overexpression on lifespan. Effects of *nhr-49* mutation and NHR-49::GFP transgene on different genetic backgrounds are depicted in this table. Data from three independent trials are shown, represented as mean lifespan in days (Mean) ± standard error of the mean (SEM). ‘n’ refers to the number of worms observed (obs) divided by total number of worms tested in the experiment. ^a^ some worms were censored from the analysis as described in methods. P values were calculated using the log rank (Mantel Cox) method. Detailed information about the strains listed here can be seen in S8 Table.
(PDF)

Table S4 Effect of reduced IIS signaling and mitochondrial electron transport chain activity on the lifespan of *nhr-49* mutants. **S4A: Effect of** ***nhr-49*** **mutation on** ***daf-2*** **mutants' longevity. S4B: RNAi lifespans.** Wild-type worms (N2) and *nhr-49* mutants were subjected to whole-life RNAi inactivation of *daf-2* (that encodes the insulin/IGF1 receptor) and genes involved in mitochondrial electron transport chain activity (*cco-1* and *cyc-1*). See methods section for experimental details. In both tables, data is represented as mean lifespan in days (Mean) ± standard error of the mean (SEM). ‘n’ refers to the number of worms observed (obs) divided by total number of worms tested in the experiment. ^a^ some worms were censored from the analysis as described in methods. ^b^ control refers to worms exposed to empty vector plasmid without an RNAi insert. P values were calculated using the log rank (Mantel Cox) method.
(PDF)

Table S5 Effect of *daf-16* mutation on the longevity of *NHR-49::GFP* worms. S5A: *daf-16* mutants do not exhibit lifespan extension upon NHR-49 overexpression. S5B: NHR-49::GFP does not rescue the longevity of *daf-16;glp-1* mutants. Data are represented as mean lifespan in days (Mean) ± standard error of the mean (SEM). ‘n’ refers to the number of worms observed (obs) divided by total number of worms tested in the experiment. ^a^ some worms were censored from the analysis as described in methods. P values were calculated using the log rank (Mantel Cox) method.
(PDF)

Table S6 Effect of RNAi inactivation of β-oxidation genes on the longevity of *glp-1* mutants and wild-type worms. *glp-1* mutants (1A) and the sterile strain, *fer-15(b26);fem-1(hc17), (1B) were subjected to ‘adult-only’ RNAi inactivation of mitochondrial **β-**oxidation genes. *fer-15(b26);fem-1(hc17)* is a temperature sensitive strain that when grown at 25°C is sterile and used as a surrogate for wild-type, N2 in lifespan assays. Data is shown as mean lifespan in days (Mean) ± standard error of the mean (SEM). ‘n’ refers to the number of worms observed (obs) divided by total number of worms tested in the experiment. ^a^ some worms were censored from the analysis as described in methods. ^b^ Empty vector control refers to worms exposed to empty vector plasmid without an RNAi insert. P values were calculated using the log rank (Mantel Cox) method.
(PDF)

Table S7 Oleic acid supplementation does not rescue the lifespan phenotypes of *nhr-49* mutants.
(PDF)

Table S8 List of strains used in this study.
(PDF)

Table S9 Q-PCR primers for genes examined in this study.
(PDF)
