# Supplementary figures and images for: Germline Signals Deploy NHR-49 to Modulate Fatty-Acid β-Oxidation and Desaturation in Somatic Tissues of C. elegans
Source: PLoS Genet. 2014 Dec 4;10(12):e1004829. doi: 10.1371/journal.pgen.1004829 (PMC4256272; doi:10.1371/journal.pgen.1004829)

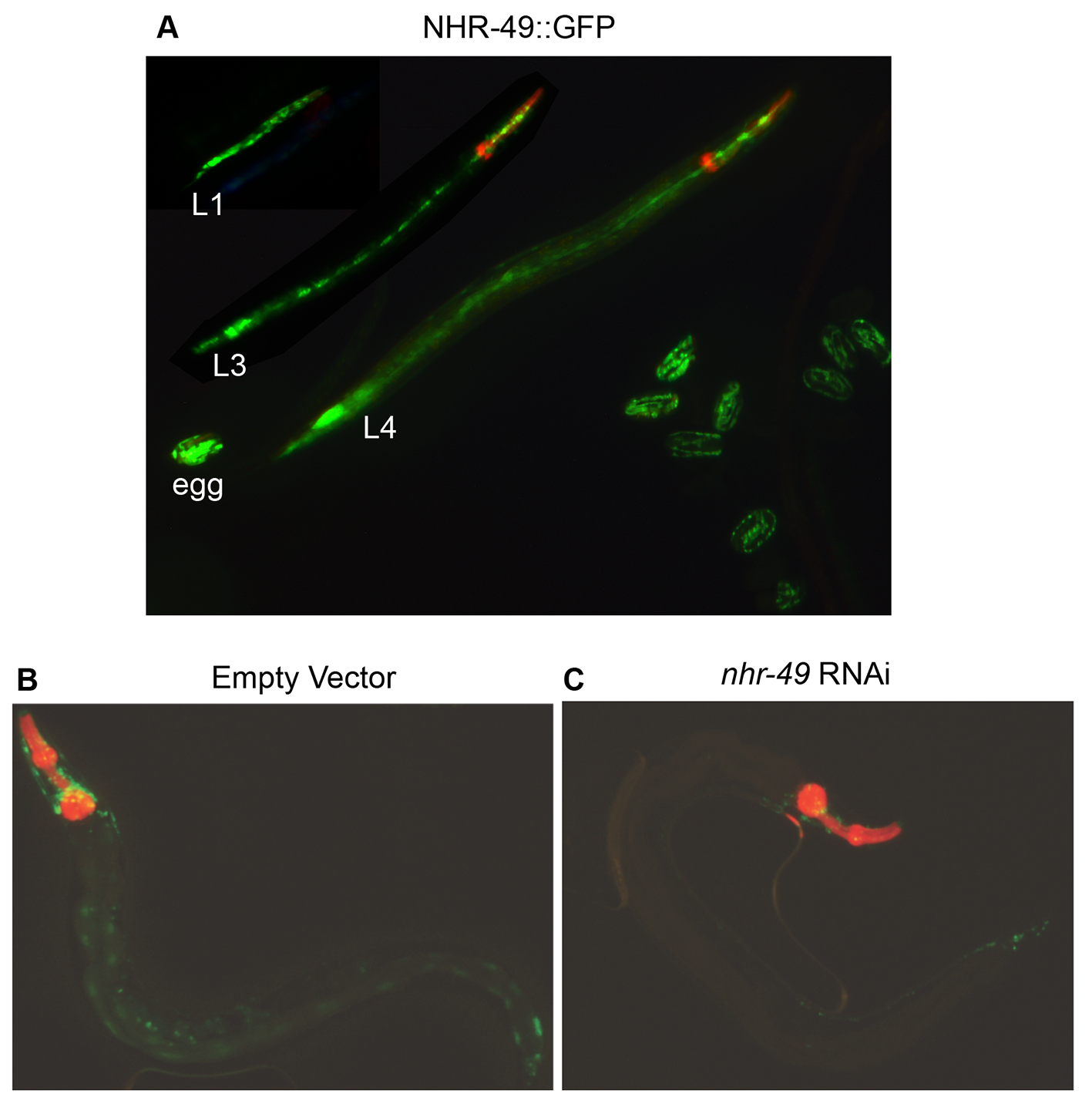

Supplement: Figure S1 — The expression of NHR-49::GFP at different developmental stages (A) and upon nhr-49 RNAi (B, C). A: Representative image of a group of NHR-49::GFP animals in different developmental stages. GFP is widely expressed in eggs, and L1-L4 larvae (L1, L3, L4 shown here). B, C: Young adults of NHR-49::GFP grown on bacteria expressing control (empty) vector (B) and bacteria expressing nhr-49 dsRNA (C). GFP expression is reduced in all somatic cells except neurons. (TIF) [file pgen.1004829.s001.tif]

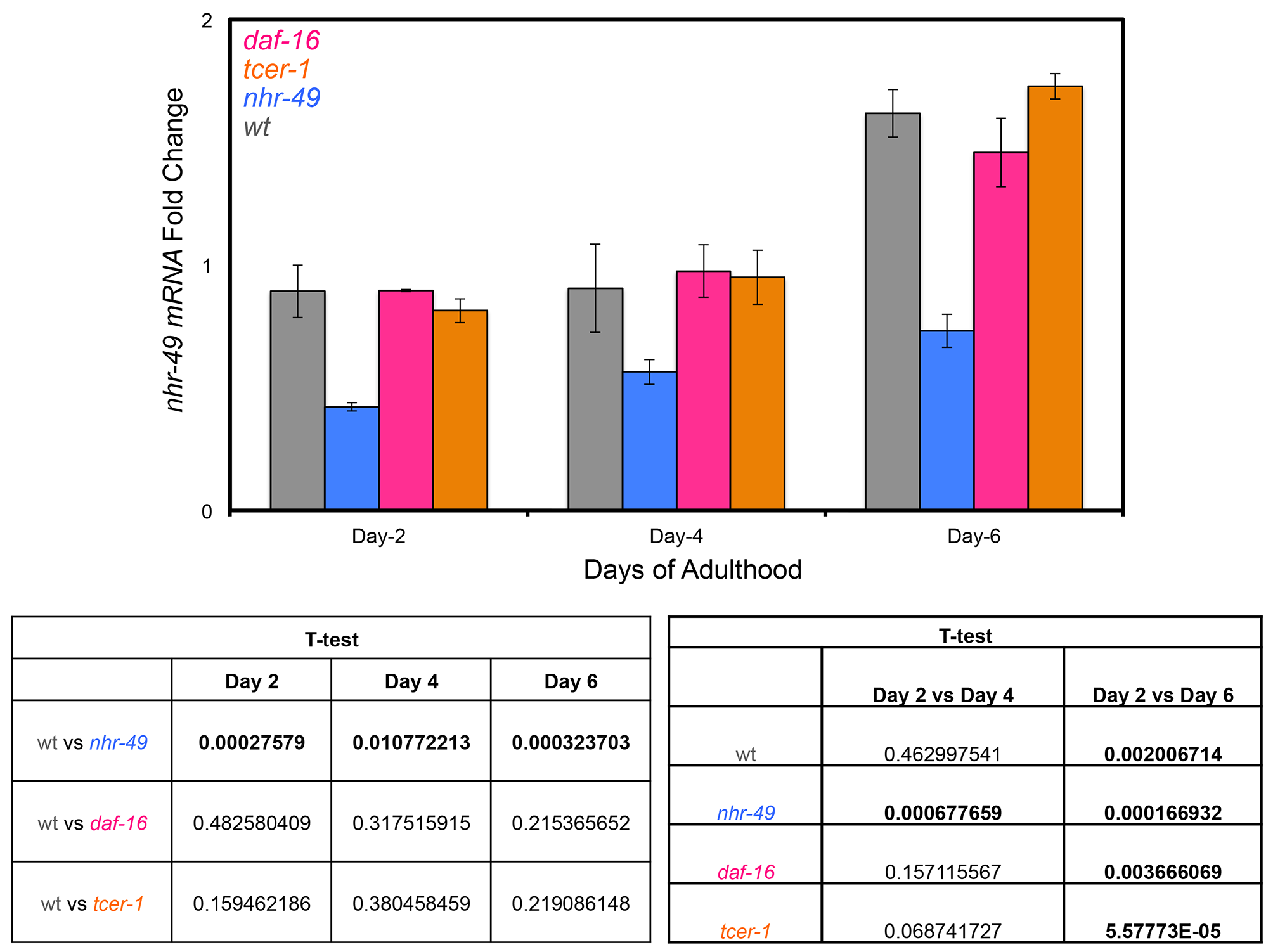

Supplement: Figure S2 — Effect of DAF-16 and TCER-1 on nhr-49 expression in normal adults. Relative mRNA levels of nhr-49 measured by Q-PCR in wild-type, N2 worms (wt, gray), nhr-49 (blue), daf-16 (red) and tcer-1 (orange) mutants on second, fourth and sixth days of adulthood. nhr-49 mutants have significantly reduced expression, but no change is observed in daf-16 or tcer-1 mutants. mRNA levels rise with age in all the strains. Statistical significances of the observed differences between different strains and ages in unpaired, two-tailed t-tests are shown in the tables. Data shown is obtained from at least three independent biological replicates. (TIF) [file pgen.1004829.s002.tif]

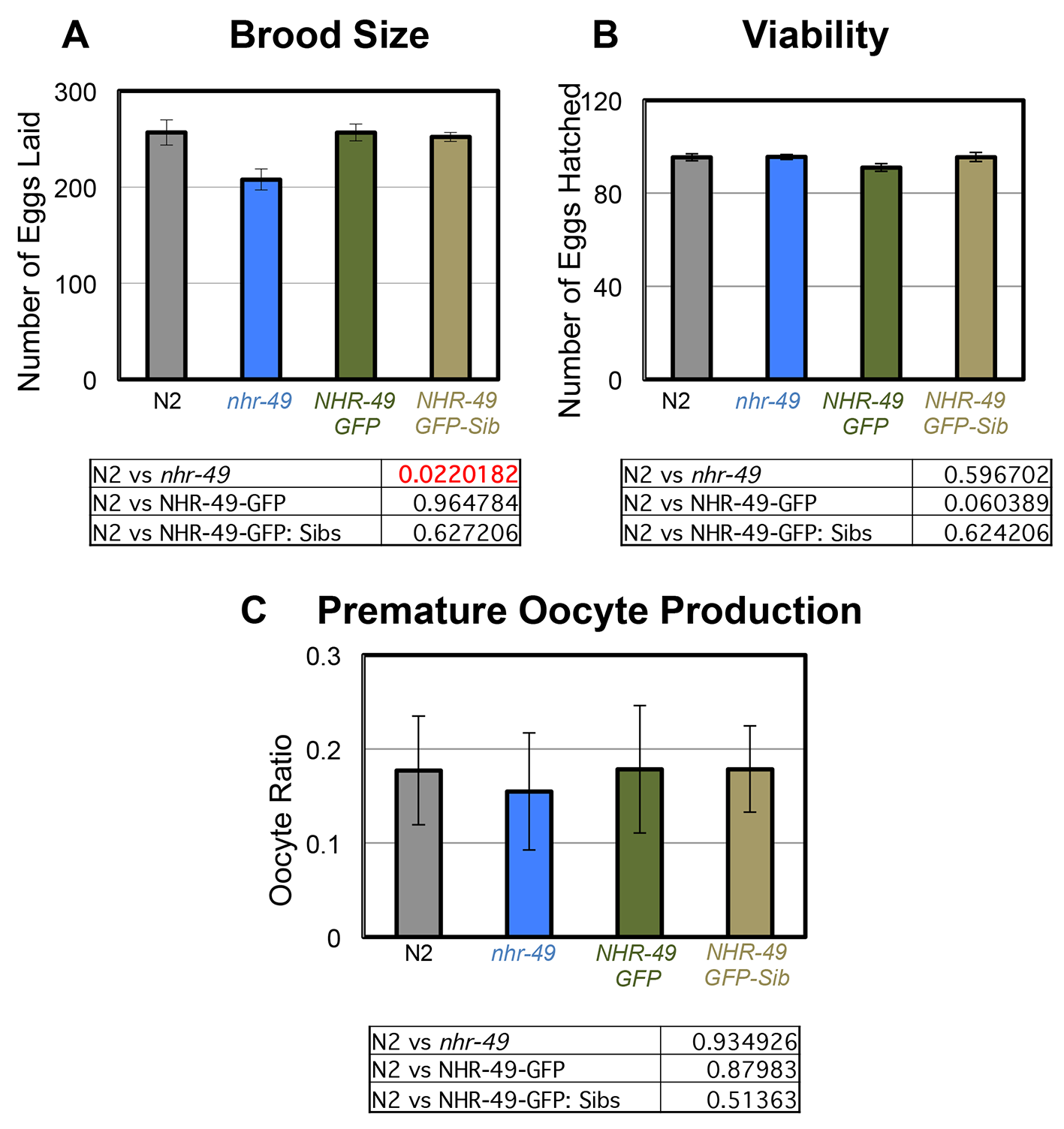

Supplement: Figure S3 — NHR-49 overexpression does not impair the reproductive health of normal, fertile animals. A-C: Bar graphs represent the comparisons of reproductive health measures between wild type (N2, gray), nhr-49 mutants (blue), NHR-49::GFP overexpressing worms (green) and the non-transgenic, control siblings of NHR-49::GFP worms (olive). A: Brood Size: total number of eggs laid during the lifetime of an animal. B: Viability: fraction of eggs laid that successfully hatch and develop into adults. C: Premature oocyte production: Older hermaphrodites and those with impaired fertility lay down unfertilized oocytes. The ratio of number of oocytes laid to the number of eggs laid (oocyte ratio) gives a measure of the fecundity of the animal. NHR-49 overexpression does not impact any of these measures negatively. Data shown here is combined from three independent biological replicates, in each of which at least ten adults were examined. Error bars display standard error of the mean, and the tables under each panel depict the statistical significance of the observed differences in an unpaired, two-tailed t-test. (TIF) [file pgen.1004829.s003.tif]

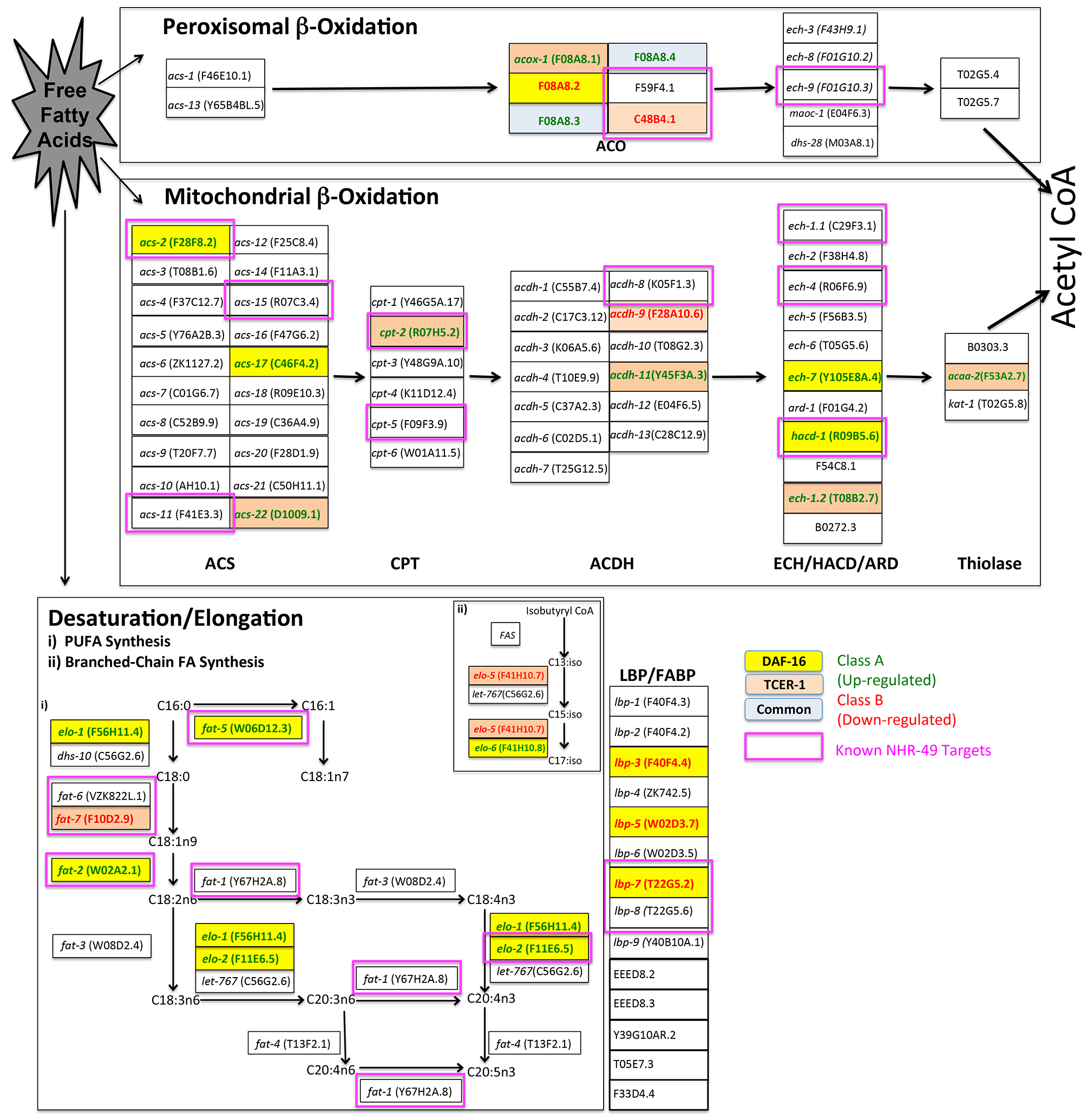

Supplement: Figure S4 — Multiple genes involved in fatty-acid oxidation, desaturation and elongation are regulated by NHR-49 and overlap with DAF-16 and TCER-1 targets in germline-less animals (modified from Amrit et al., manuscript in preparation). The genes predicted to function in different steps of peroxisomal and mitochondrial β-oxidation (top and middle panels) and fatty-acid desaturation and elongation (bottom panel) are depicted in individual rectangles. Cosmid numbers are provided in brackets next to each gene. Genes identified previously as NHR-49 targets [36], [37], [46] are highlighted with purple rectangles. They show a significant overlap with genes identified as DAF-16 and TCER-1 targets in an RNA-Seq study (Amrit et al., manuscript in preparation) represented here as colored rectangles: DAF-16 targets (yellow), TCER-1 targets (cream) and joint targets (blue). Genes up-regulated by these proteins are shown in green font and those repressed are in red font. The enzymes produced by β-oxidation genes are depicted under each category. ACS: acyl CoA synthetase; CPT: carnitine palmitoyl transferase; ACDH: acyl CoA dehydrogenase; ECH: enoyl CoA hydratase; HACD: hydroxyl acyl CoA dehydrogenase. Free fatty acids are broken down to acetyl CoA moieties by β-oxidation. They can also undergo desaturation and elongation to give rise to larger, unsaturated species that can be stored as triglycerides or incorporated into membranes. Genes involved in the poly-unsaturated fatty acid (PUFA) synthesis (i) and branched chain fatty acid synthesis (ii) pathways are shown here. In addition, lipid binding proteins (LBP) and fatty acid binding proteins (FABP) that transport fatty acids and are important for these processes are also included. (TIF) [file pgen.1004829.s004.tif]

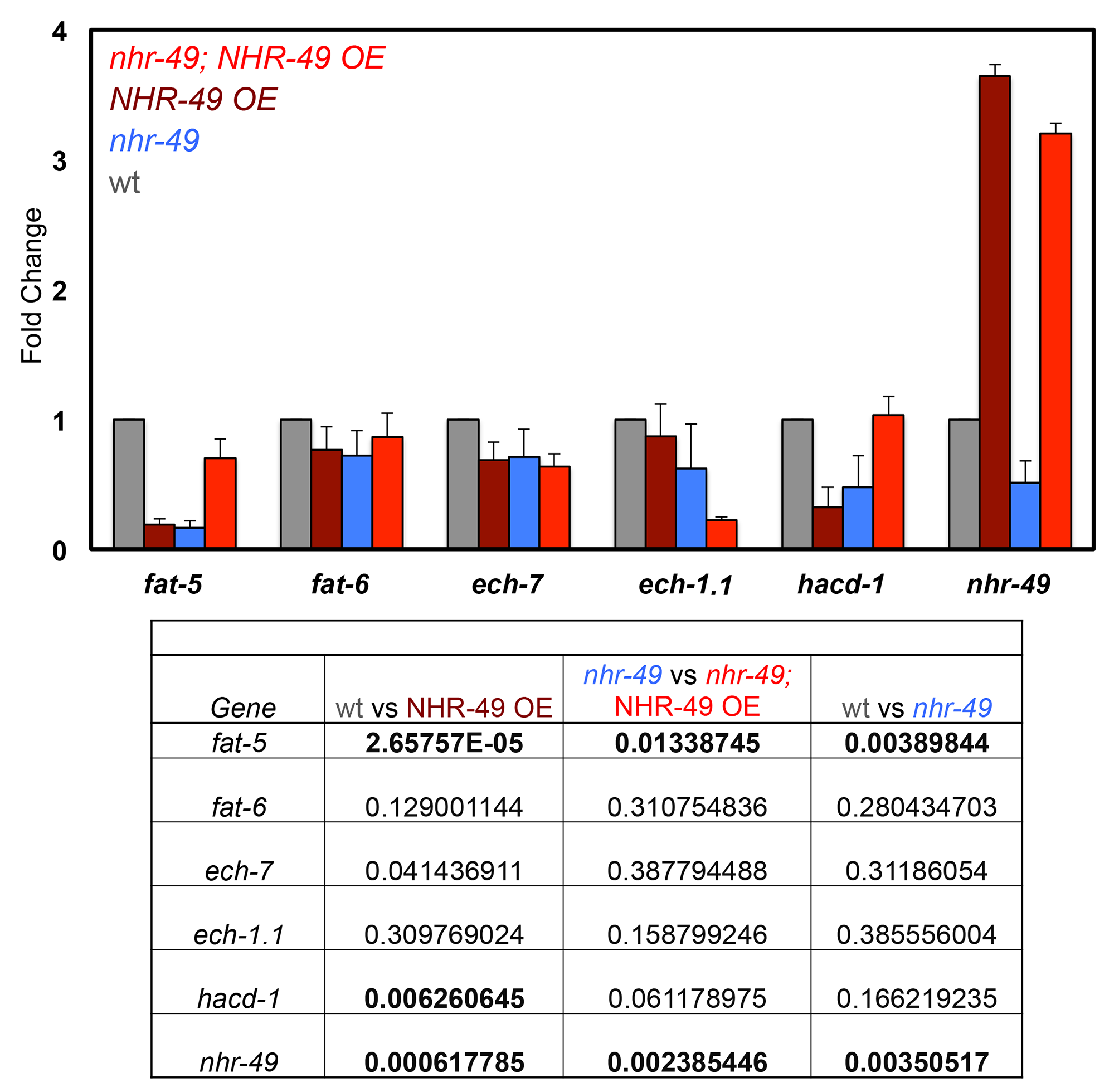

Supplement: Figure S5 — mRNA levels of β-oxidation and desaturation genes up-regulated in glp-1 mutants in an NHR-49-dependent manner examined in nhr-49 mutants and NHR-49::GFP strains. Relative mRNA levels measured by Q-PCR in day 2 adults of wild-type, N2 worms (wt, gray), nhr-49 mutants (blue), NHR-49::GFP (NHR-49 OE, maroon) and nhr-49;NHR-49::GFP (nhr-49;NHR-49 OE, red) strains. nhr-49 level was reduced in nhr-49 mutants, and elevated in the NHR-49 OE strains, as expected. mRNA levels of the target genes were not elevated. Statistical significances of the observed differences between different strains in unpaired, two-tailed t-tests are shown in the table. Data shown is obtained from at least three independent biological replicates. (TIF) [file pgen.1004829.s005.tif]

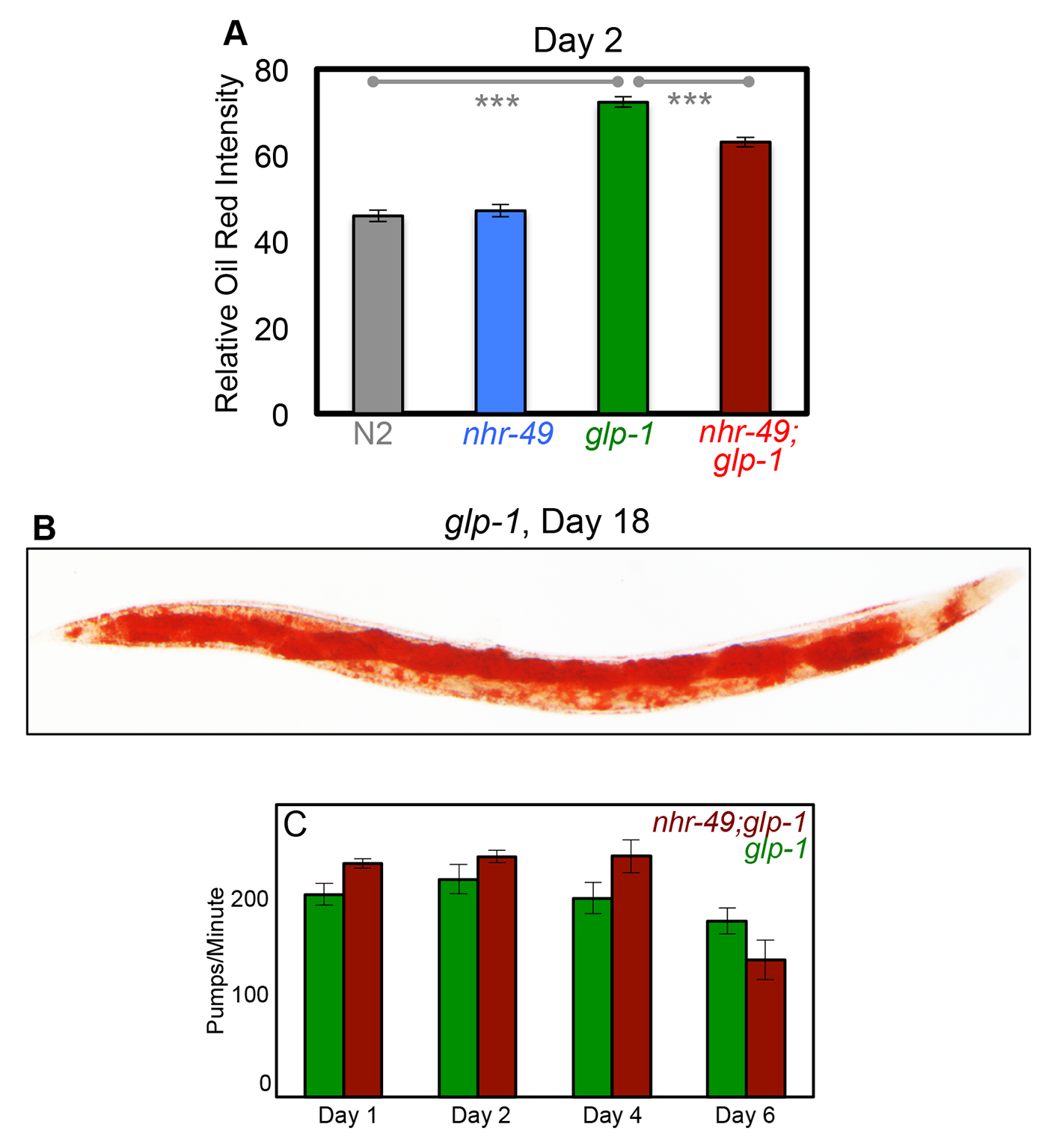

Supplement: Figure S6 — glp-1 mutants exhibit high fat levels for significant fraction of adulthood. A: Bar graphs represent the quantification of fat levels estimated based on the intensity of ORO staining in day-2 wild type (N2), nhr-49 (blue), glp-1 (green) and nhr-49;glp-1 (red) animals. Both glp-1 and glp-1;nhr-49 mutants show significant increase in staining compared to N2 and nhr-49. nhr-49;glp-1 fat levels are modestly but significantly lesser than glp-1 adults. Error bars display standard error of the mean, and asterisks depict the statistical significance of the observed differences in an unpaired, two-tailed t-test with P<0.0001 (***). Data shown here is obtained from 3 independent biological replicates in which all four strains were tested simultaneously. B: Representative image of ORO staining in day 18 glp-1 adult. glp-1 mutants continued to show high fat accumulation in intestinal cells on day 18 and even up to day 30 (not shown) of adulthood (by comparison, nhr-49; glp-1 mutants lost almost all intestinal fat by day 8 and the entire population perished by day 15). C: nhr-49 ; glp-1 mutants exhibit normal rate of pharyngeal pumping. Number of pharyngeal pumps per minute (Y-axis) were compared between glp-1 and nhr-49;glp-1 adults on days 1, 2, 4 and 6 (X-axis). No significant difference was observed at any time point. (TIF) [file pgen.1004829.s006.tif]

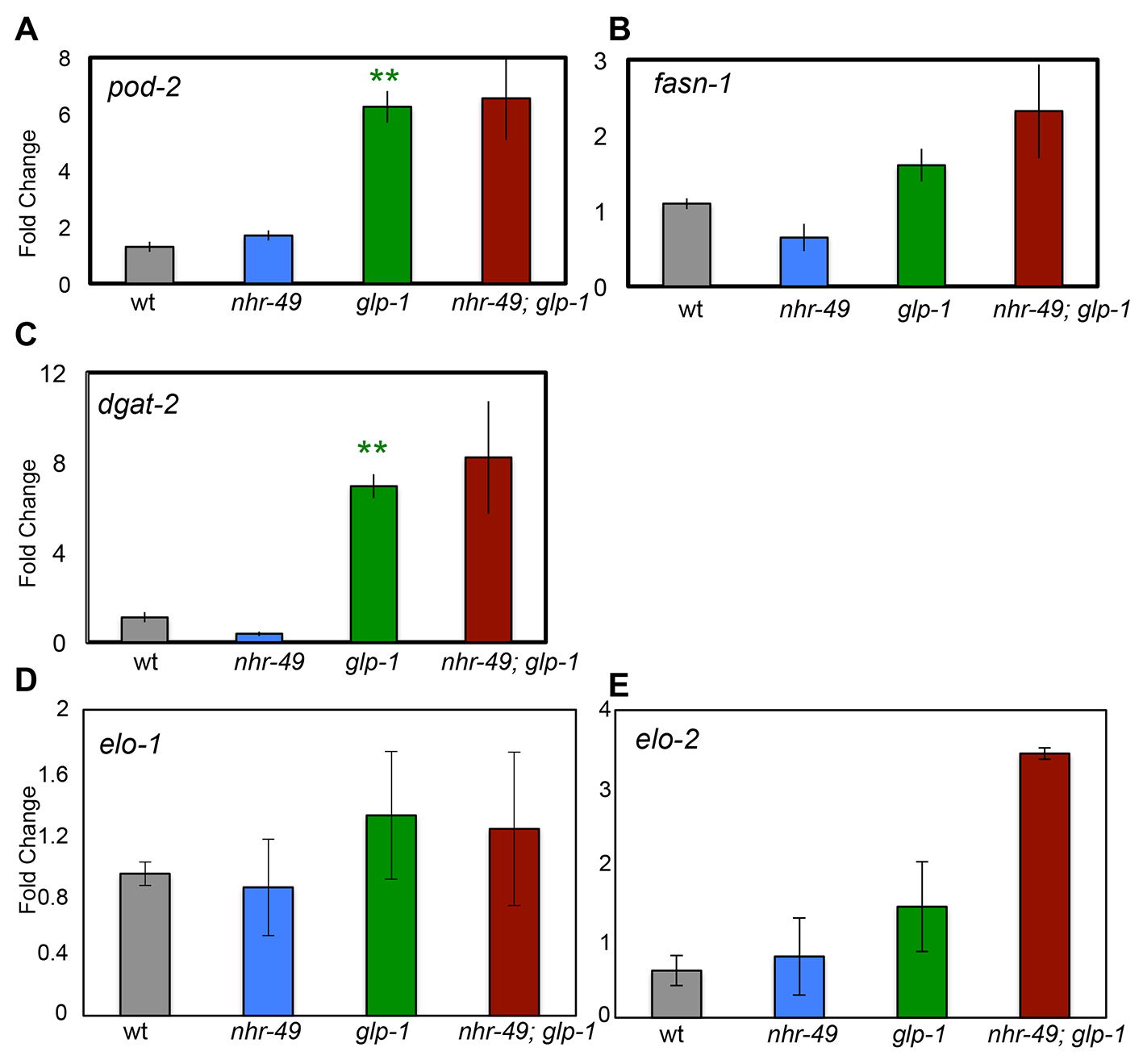

Supplement: Figure S7 — The effect of nhr-49 mutation on expression genes involved in initiation of fatty-acid synthesis and elongation. A–C: Relative mRNA levels measured by Q-PCR of key genes involved in initiation of fat synthesis, pod-2 (A) and fasn-1 (B), compared between day 2 wild-type worms (wt, gray), nhr-49 (blue), glp-1 (green) and nhr-49;glp-1 (maroon) mutants. In C, the expression of dgat-2 that encodes a diacylglycerol acyl transferase (DGAT) enzyme is probed. DGAT-2 is a rate-limiting enzyme needed for diglyceride (DAG) to triglyceride (TAG) conversion. D, E: Relative mRNA expression of elongase-encoding genes, elo-1 and elo-2, compared by Q-PCR between the same strains. In all panels, the X-axis shows the strains and Y-axis the fold change in expression. Error bars display standard error of the mean and asterisks depict the statistical significance of the observed differences in an unpaired, two-tailed t-test with P values 0.05 (*) and 0.005 (**). Data shown is obtained from at least three independent biological replicates. (TIF) [file pgen.1004829.s007.tif]

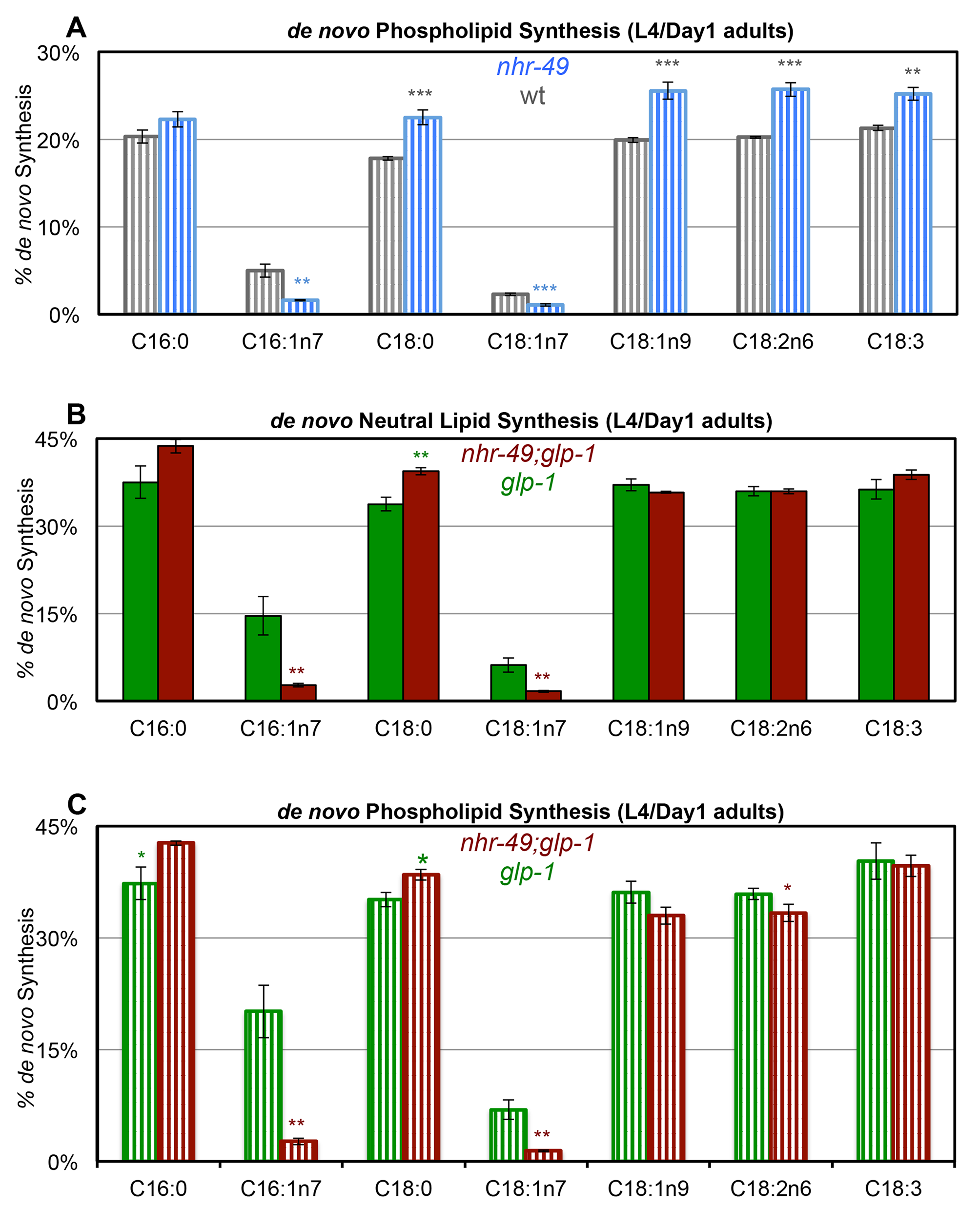

Supplement: Figure S8 — de novo fatty-acid synthesis is impaired in late L4/early day 1 adults carrying the nhr-49 mutation. Using a 13C isotope fatty-acid labeling assay, de novo fat synthesis and dietary fat absorption were compared between late L4/early day adults of wild-type, N2 (wt) worms and nhr-49 single mutants (A) and age-matched glp-1 and nhr-49;glp-1 mutants (B, C). Neutral lipid data is shown in solid bars (B) and phospholipid data in striped bars (A, C). Individual fatty-acid species are represented on the X-axis and relative synthesis levels are on the Y-axis. Graphs in all panels were obtained by combining data from three independent biological replicates. Asterisks show the statistical significance of the observed differences in an unpaired, two-tailed t-test with P values 0.05 (*), 0.005 (**) and <0.0001 (***). The color of the asterisk denotes the strain showing the observed reduction. (TIF) [file pgen.1004829.s008.tif]

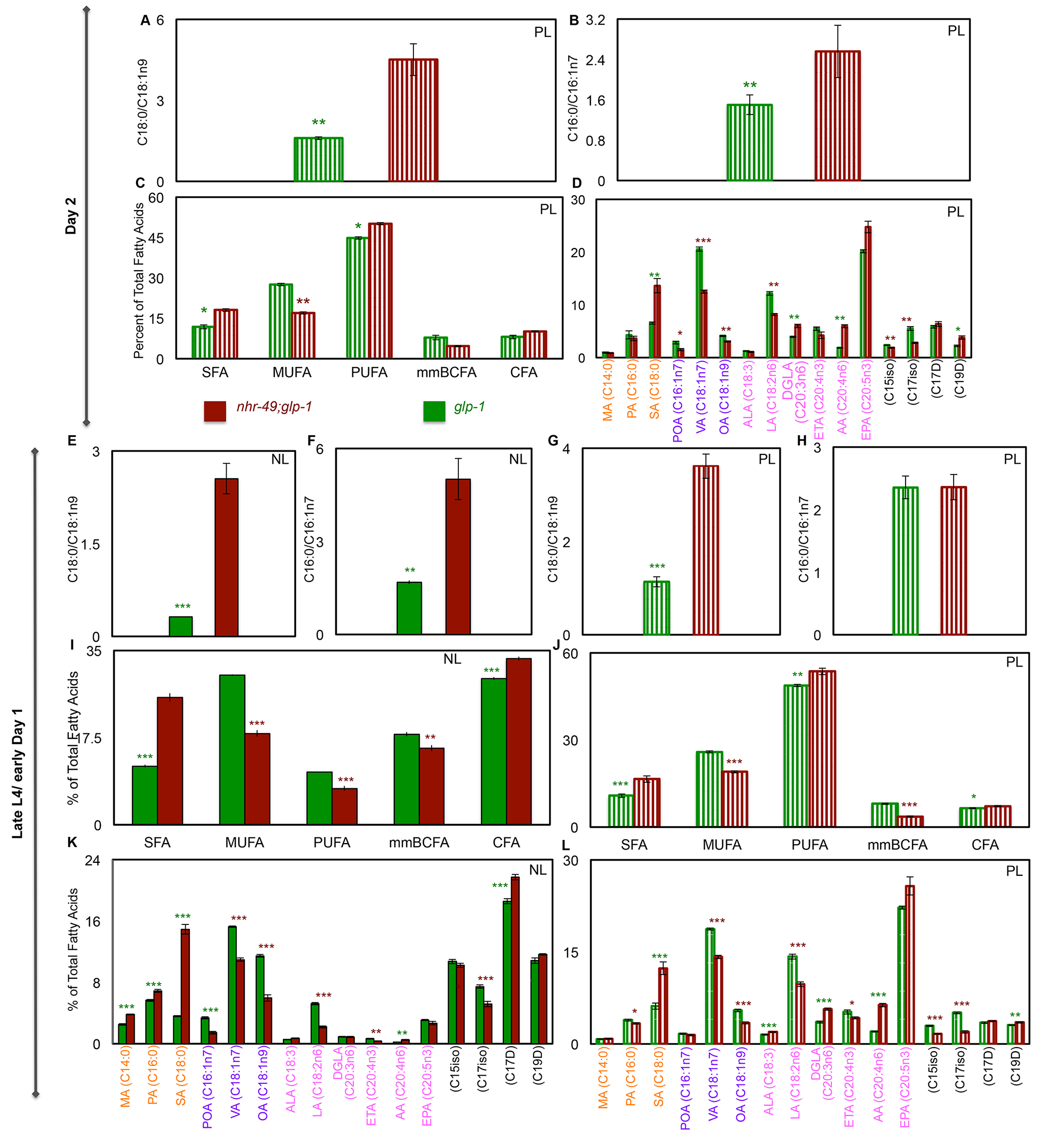

Supplement: Figure S9 — Effect of nhr-49 mutation on fatty-acid composition in germline-less animals. Comparisons of fatty-acid profiles between glp-1 (green) and nhr-49;glp-1 (maroon) mutants' phospholipids (PL, striped bars) isolated from day 2 adults (A–D) and neutral lipids (NL, solid bars) as well as phospholipids isolated from L4/early day 1 adults (E–L). A, E, G: SA:OA ratio is increased in nhr-49;glp-1 mutants in both lipid populations and at both ages. B, F, H: PA:POA ratio is increased in nhr-49;glp-1 mutants at both ages in the neutral lipid population (B, F), but not in the phospholipids of late L4/early day 1 adults (H). C, D, I–L: Overall MUFA levels are reduced and SFAs elevated in nhr-49 ; glp-1 mutants. In the phospholipid fraction of day 2 adults (C, D) and in both the neutral and and phospholipid fractions of late L4/early day 1 adults (I–L), nhr-49;glp-1 mutants showed a significant reduction in the overall level of MUFAs, whereas SFA content was elevated. Similar overall profiles were observed at both stages with some exceptions. PUFA levels were significantly increased in the phospholipids of nhr-49;glp-1 mutants at both ages, whereas they were reduced in the neutral lipid fractions at both ages (the effect did not achieve statistical significance for day 2 adults, Fig. 6H). Comparisons of individual fatty acid levels are shown in D, K and L. In K and L, SFAs are labeled in orange (MA: myristic acid), MUFAs in purple (VA: vaccenic acid) and PUFAs in pink (ALA: α-linolenic acid; LA: linoleic acid; DGLA: dihomo γ-linolenic acid; ETA: eicosatetraenoic acid; AA: arachidonic acid; EPA: eicosapentaenoic acid). Black labels mono-methyl branched chain fatty acids (mmBCFA) and cyclopropane fatty acids (CFA). Y-axes indicate the percent of total fatty-acid pool contributed by each of the species indicated on the X-axes. Asterisks show the statistical significance of the observed differences in an unpaired, two-tailed t-test with P values 0.05 (*), 0.005 (**) and <0.0001 (***). [file pgen.1004829.s009.tif]

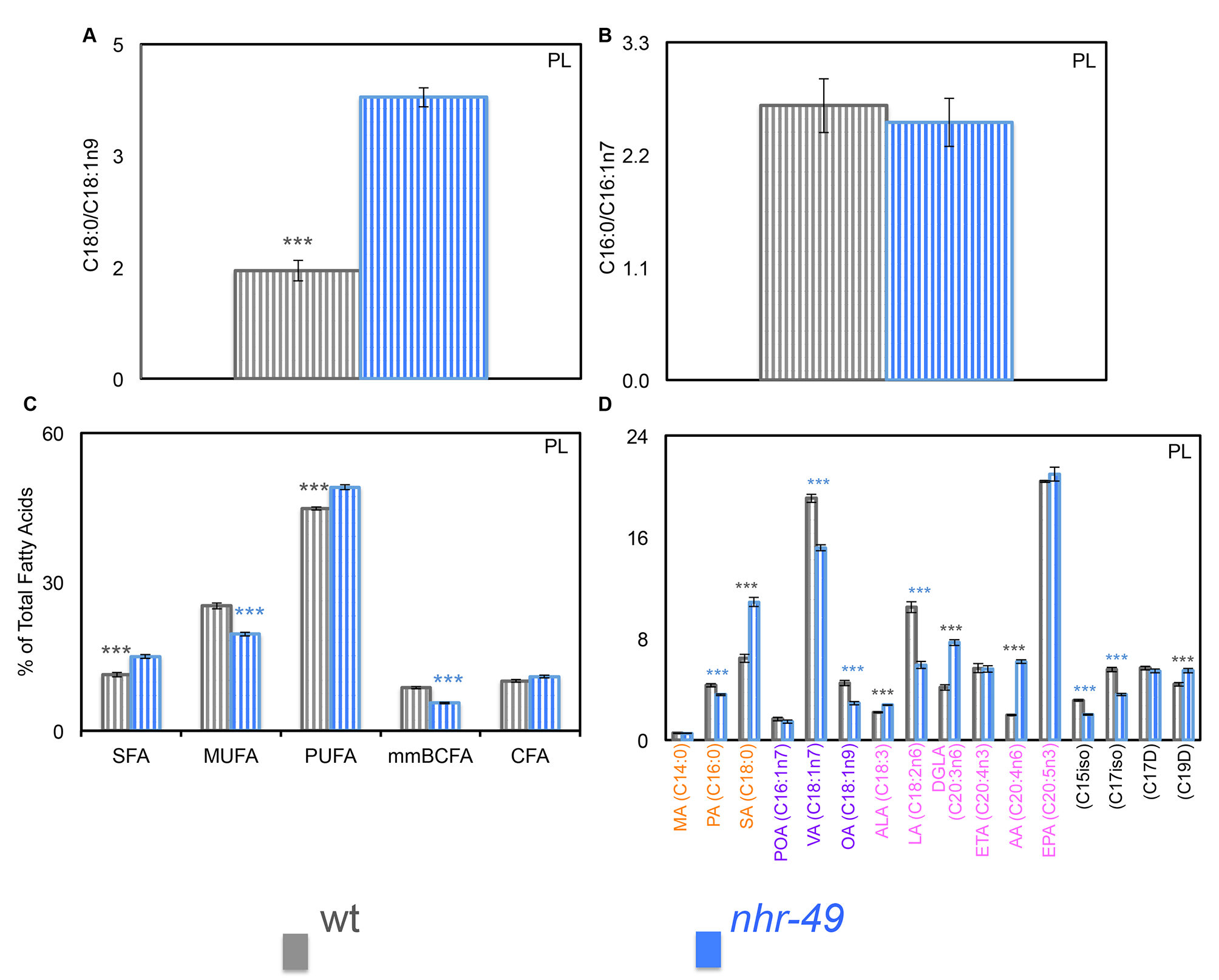

Supplement: Figure S10 — Comparison of fatty-acid composition of phospholipids isolated from late L4/early day 1 nhr-49 mutants and age-matched wild-type worms. A, B: SA:OA ratio is elevated (A) but not PA:POA ratio (B) in nhr-49 mutants. C, D: nhr-49 mutants show increased overall SFAs and reduced MUFAs. Levels of PUFAs and mmBCFAs are altered as well (C). Variations in individual fatty acid levels are shown in D. Graphs in all panels were obtained by combining data from three independent biological replicates. Asterisks show the statistical significance of the observed differences in an unpaired, two-tailed t-test with P values 0.05 (*), 0.005 (**) and <0.0001 (***).The color of the asterisk denotes the strain showing the observed reduction. (TIF) [file pgen.1004829.s010.tif]

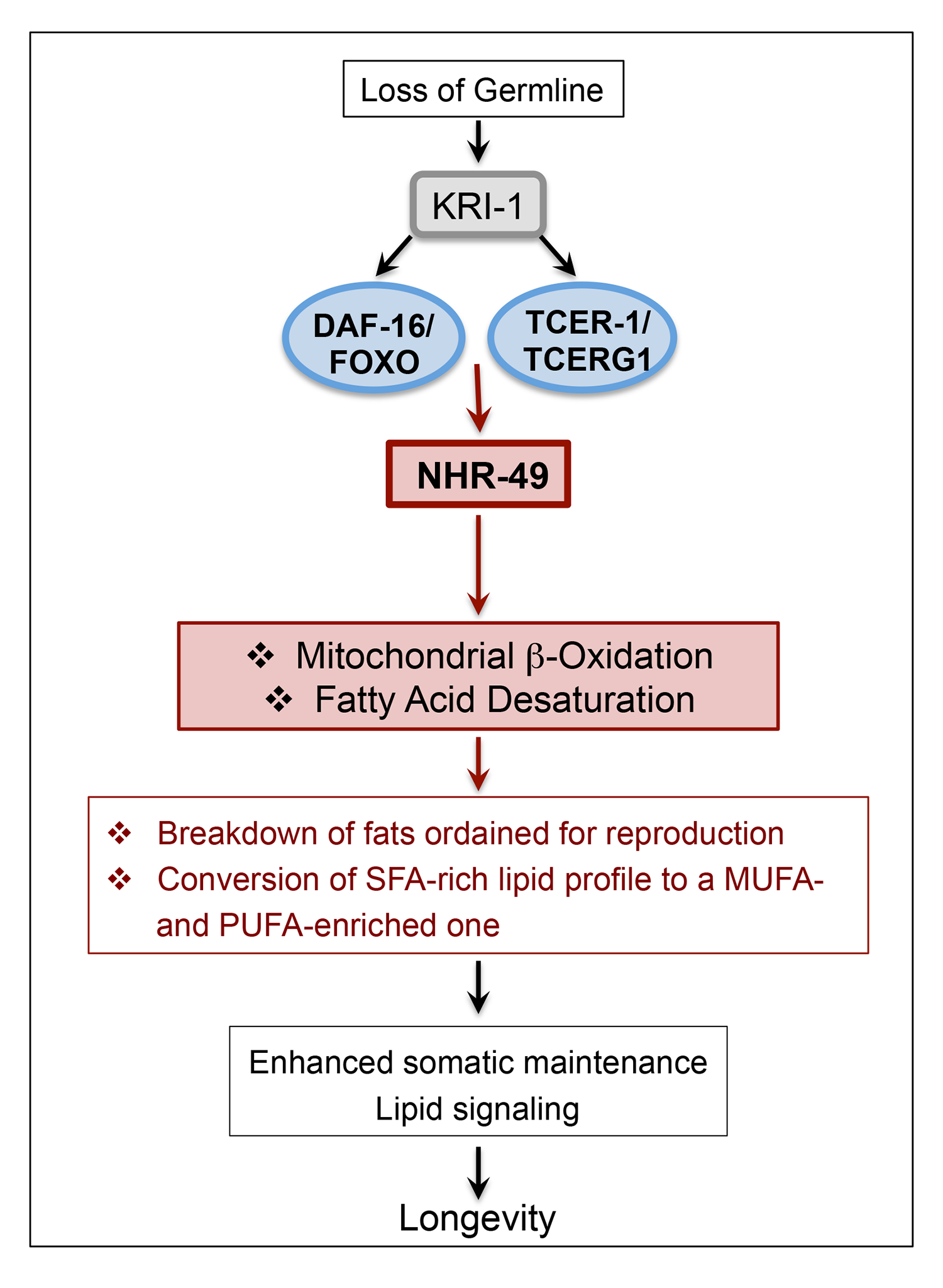

Supplement: Figure S11 — Schematic representation of the proposed model to explain NHR-49 function in promoting the longevity of germline-less animals. Following germline loss, NHR-49 is up-regulated by the joint activity of DAF-16 and TCER-1. NHR-49, in turn, mediates the up-regulation of genes involved in fatty-acid β-oxidation and desaturation. The synchronized enhancement of these processes allows the animal to adapt to loss of fertility and orchestrate a lipid-homeostasis profile that supports longevity. Red arrows indicate the steps for which evidence is provided in this study. (TIF) [file pgen.1004829.s011.tif]

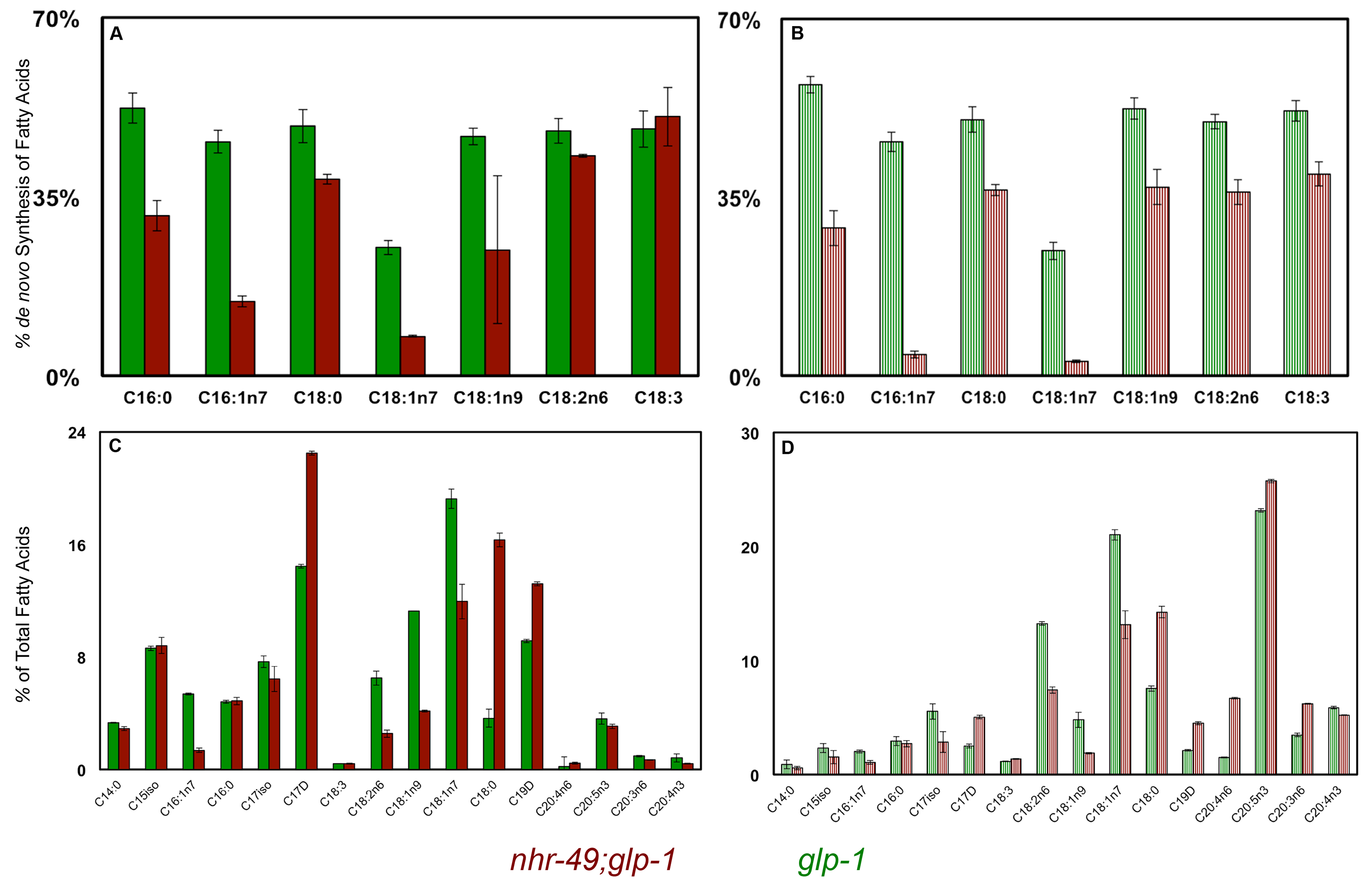

Supplement: Figure S12 — de novo lipid synthesis and fatty-acid comparison between day 2 glp-1 adults and nhr-49;glp-1 mutants harvested after a – hour delay. A, B: de novo fat synthesis and dietary fat absorption data obtained through 13C isotope fatty-acid labeling assay compared between glp-1 (green) and nhr-49;glp-1 (maroon) (B, C). Neutral lipid data is shown in solid bars (A) and phospholipid data in striped bars (B). Individual fatty-acid species are represented on the X-axis and relative synthesis levels are on the Y-axis. C, D: Comparisons of fatty-acid profiles between the same strains through GC/MS analysis. Please note that the order of representation of individual fatty acids in C and D is different from that used in other graphs in the article. (TIF) [file pgen.1004829.s012.tif]
