# Supplementary material for: Germline Signals Deploy NHR-49 to Modulate Fatty-Acid β-Oxidation and Desaturation in Somatic Tissues of C. elegans
Source: PLoS Genet. 2014 Dec 4;10(12):e1004829. doi: 10.1371/journal.pgen.1004829 (PMC4256272; doi:10.1371/journal.pgen.1004829)
Supplement: Table S1 — Retests of select ‘nhr’ RNAi clones found to suppress the up-regulation of GFP in glp-1;Pstdh-1::GFP worms in RNAi screen. RNAi clones that were found to impair GFP expression in glp-1;Pstdh-1/dod-8::gfp day 2 adults were retested thrice by one (retest #3) or two independent observers. The results are tabulated along with the identities of the genes and their respective cosmid numbers. MV refers to an RNAi clone obtained from the Vidal collection [39]. All other clones were from the Ahringer library [40]. (PDF) [file pgen.1004829.s013.pdf]

Ratnappan et al., REVISED. Table S1: Retests of select clones found to suppress the up-regulation of *glp-1*; *Pstdh-1::GFP* in RNAi screen (whole-life RNAi)

		Retest #1			Retest #2		Retest #3	
**#**	**Gene (cosmid #)**	**Observer 1**	**Observer 2**	**Comments**	**Observer 1**	**Comments**	**Observer 2**	**Comments**
1	*nhr-17 (C02B4.2)*	*	*		X	no effect	*	mild suppressor
2	*nhr-31 (C26B2.3)*	**	**	strong suppressor	*		*	
3	*nhr-41 (Y104H12A.1)*	not screened	*	mild suppressor	X	no effect	XX	higher expression
4	*nhr-49 (K10C3.6)*	*	**	strong suppressor	XX	higher GFP	**	strong suppressor
5	*nhr-49 (K10C3.6) MV clone*	*	*		X	no effect	*	strong suppressor
6	*nhr-54 (F36D3.2)*	XX	*	no effect or higher GFP	*	mild suppression	*	mild suppressor
7	*nhr-60 (F57A10.5)*	**	**	strong suppressor	**	strong suppressor	X	no effect
8	*nhr-61 (W01D2.2)*	**	**		**		**	wrong clone; discarded
9	*nhr-71 (K11E4.5)*	*	*		*		**	originally identified as *nhr-33*
10	*nhr-80 (H10E21.3)*	**	**		*		**	
11	*nhr-81 (C47F8.8)*	**	**	strong suppressor	**	strong suppressor	**	strong suppressor
12	*nhr-87 (Y41D4B.7)*	**	**	strong suppressor	**	strong suppressor	*	
13	*nhr-87 (Y41D4B.7) MV clone*	**	**	strong suppressor	*	mild suppressor	XX	higher expression
14	*nhr-122 (Y41D4B.9)*	*	X	no effect	XX	higher GFP	XX	higher expression
15	*nhr-164 (C41G6.5)*	*	**		X	no effect	XX	higher expression
16	*nhr-199 (K06B4.10)*	XX	XX	higher expression	XX	medium high	XX	both as 'no effect'
17	*nhr-209 (R07B7.16)*	**	**	strong suppressor		censored	**	low anterior expression
18	*nhr-212 (T01G6.5)*			censored	**	strong suppressor	**	strong suppressor
19	*nhr-213 (T06C12.13)*	X	**	strong suppressor for some		censored	*	
20	*nhr-255 (ZK678.2)*	*	**	low anterior expression	X	no effect	*	low anterior expression
21	*nhr-274 (Y41D4B.21)*	**	**	arrested	**	arrested	**	arrested
22	*nhr-274 (Y41D4B.21) MV clone*	**	**		**		**	

** strong suppression of GFP expression
* mild suppression of GFP expression

XX increased GFP expression (Discrepancy with screen)
X no effect on GFP levels or slight increase (Discrepancy from screen)

(grey shading) Clones did not reproducibly effect GFP levels or shorten *glp-1* longevity
