# Supplementary material for: Germline Signals Deploy NHR-49 to Modulate Fatty-Acid β-Oxidation and Desaturation in Somatic Tissues of C. elegans
Source: PLoS Genet. 2014 Dec 4;10(12):e1004829. doi: 10.1371/journal.pgen.1004829 (PMC4256272; doi:10.1371/journal.pgen.1004829)
Supplement: Table S3 — Effect of NHR-49 loss-of-function and overexpression on lifespan. Effects of nhr-49 mutation and NHR-49::GFP transgene on different genetic backgrounds are depicted in this table. Data from three independent trials are shown, represented as mean lifespan in days (Mean) ± standard error of the mean (SEM). ‘n’ refers to the number of worms observed (obs) divided by total number of worms tested in the experiment. a some worms were censored from the analysis as described in methods. P values were calculated using the log rank (Mantel Cox) method. Detailed information about the strains listed here can be seen in S8 Table. (PDF) [file pgen.1004829.s015.pdf]

**Ratnappan et al., REVISED. Table S3: Effect of NHR-49 loss-of-function and overexpression on lifespan**

| | | Trial 1 | | | | Trial 2 | | | | Trial 3 | | | |
|---|---|---|---|---|---|---|---|---|---|---|---|---|---|
| # | Strain | n = 0bs/Tot[a] | Mean ± SEM | P (*vs.* N2) | P (*vs.* other strains) | n = 0bs/Tot[a] | Mean ± SEM | P (*vs.* N2) | P (*vs.* other strains) | n = 0bs/Tot[a] | Mean ± SEM | P (*vs.* N2) | P (*vs.* other strains) |
| 1 | *N2* | 75/98 | 21.6 ± 0.1 | | | 81/92 | 22.8 ± 0.2 | | | 89/110 | 22.6 ± 0.4 | | |
| 2 | AGP12a<br>*nhr-49(-)* | 89/100 | 14.4 ± 0.2 | <0.0001 | | 83/88 | 13.7 ± 0.1 | <0.0001 | | 59/75 | 15.6 ± 0.2 | <0.0001 | |
| 3 | AGP24f<br>*Pnhr-49::nhr-49::GFP* | 47/84 | 26.0 ± 0.4 | <0.0001 | <0.0001 (*vs. nhr-49*)<br><0.0001 (*vs.* GFP-ve siblings) | 94/99 | 23.1 ± 0.3 | 0.54 | <0.0001 (*vs. nhr-49*)<br>0.16 (*vs* GFP-ve siblings) | 58/113 | 25.9 ± 0.3 | 0.001 | (<0.0001 *vs* GFP-ve sibs) |
| 4 | *AGP 24f GFP-ve siblings* | 83/102 | 21.3 ± 0.4 | 0.82 | <0.0001 | 87/100 | 20.3 ± 0.6 | 0.23 | <0.0001 (*vs nhr-49*) | 82/137 | 22.2 ± 0.3 | 0.9 | |
| 5 | AGP33<br>*nhr-49; Pnhr-49::nhr-49::GFP* | 74/99 | 24.2 ± 0.2 | 0.001 | <0.0001 (*vs. nhr-49*)<br><0.0001 (*vs.* GFP-ve siblings) | 79/101 | 25.1 ± 0.3 | <0.0001 | <0.0001 (*vs. nhr-49*)<br><0.0001 (*vs.* GFP-ve siblings) | | | | |
| 6 | *AGP33 GFP-ve siblings* | 95/100 | 14.1 ± 0.2 | <0.0001 | 0.41 (*vs. nhr-49*) | 93/98 | 12.4 ± 0.1 | <0.0001 | 0.02 (*vs. nhr-49*) | | | | |
| 7 | CF1903<br>*glp1(-)* | 94/101 | 31.0 ± 0.5 | | | 74/104 | 35.8 ± 0.3 | | | 82/83 | 34.6 ± 0.02 | | |
| 8 | AGP22<br>*nhr-49(-);glp1(-)* | 95/97 | 14.1 ± 0.1 | <0.0001 | <0.0001 (*vs. glp-1*) | 104/106 | 17.9 ± 0.2 | <0.0001 | <0.0001 (*vs. glp-1*) | 91/91 | 13.6 ± 0.1 | <0.0001 | <0.0001 (*vs. glp-1*) |
| 9 | AGP25<br>*glp-1(-); Pnhr-49::nhr-49::GFP* | 92/97 | 35.3 + 0.5 | <0.0001 | <0.0001 (*vs. nhr-49; glp-1*)<br>0.46 (*vs.* GFP-ve siblings)<br>0.001 (vs. *glp-1*) | 97/98 | 41.0 + 0.7 | <0.0001 | <0.0001 (*vs. nhr-49; glp-1*)<br><0.0001 (*vs.* GFP-ve siblings)<br>0.002 (*vs. glp-1*) | | | | |
| 10 | AGP25 GFP-ve siblings | 98/101 | 34.7 + 0.4 | <0.0001 | <0.0001 (*vs. nhr-49; glp-1*)<br>0.003 (*vs. glp-1*) | 104/119 | 31.9 + 0.7 | <0.0001 | <0.0001 (*vs. nhr-49; glp-1*)<br>0.02 (*vs. glp-1*) | | | | |
| 11 | AGP34a<br>*nhr-49(-); glp-1(-); Pnhr-49::nhr-49::GFP* | 74/104 | 30.2 + 0.5 | <0.0001 | 0.73 (*vs glp-1*)<br><0.0001 (*vs. nhr-49; glp-1*)<br><0.0001 (*vs.* GFP-ve siblings) | 58/102 | 34.1 + 0.4 | | 0.84 (*vs. glp-1*)<br><0.0001 (*vs. nhr-49; glp-1*)<br><0.0001 (*vs.* GFP-ve siblings) | | | | |
| 12 | AGP34a GFP-ve siblings | 86/98 | 17.0 ± 0.4 | <0.0001 | 0.0007 (*vs. nhr-49; glp-1*) | 101/107 | 18.4 ± 0.2 | <0.0001 | 0.28 (*vs. nhr-49; glp-1*) | | | | |
| 13 | AGP28c<br>*Pnhr-49::nhr-49::GFP* | 64/97 | 30.0 ± 0.2 | <0.0001 | <0.0001 (*vs. nhr-49*)<br><0.0001 (*vs.* GFP-ve siblings) | | | | | 61/96 | 28.2 ± 0.3 | <0.0001 | <0.0001 (*vs. nhr-49*)<br><0.0001 (*vs.* GFP-ve siblings) |
| 14 | *AGP 28c GFP-ve siblings* | 81/98 | 22.0 ± 0.2 | 0.34 | <0.0001 (*vs. nhr-49*) | | | | | 56/66 | 21.7 ± 0.4 | 0.61 | <0.0001 (*vs. nhr-49*) |
| 15 | AGP30b<br>*nhr-49; Pnhr-49::nhr-49::GFP* | 69/85 | 22.7 ± 0.3 | 0.11 | <0.0001 (*vs. nhr-49*)<br><0.0001 (*vs.* GFP-ve siblings) | | | | | 40/100 | 24.7 ± 0.6 | 0.02 | <0.0001 (*vs. nhr-49*)<br><0.0001 (*vs.* GFP-ve siblings) |
| 16 | *AGP30b GFP-ve siblings* | 84/88 | 14.1 ± 0.2 | <0.0001 | 0.5 (*vs. nhr-49*) | | | | | 56/80 | 14.3 ± 0.1 | <0.0001 | 0.08 (*vs. nhr-49*) |
| 17 | AGP29a<br>*glp-1(-); Pnhr-49::nhr-49::GFP* | | | | | | | | | 64/83 | 35.4 ± 0.3 | 0.91 | <0.0001 (*vs. nhr-49; glp-1*)<br>0.0027 (*vs.* GFP-ve siblings) |
| 18 | AGP29a GFP-ve siblings | | | | | | | | | 84/87 | 30.1 ± 0.3 | 0.001 | <0.0001 (*vs. nhr-49; glp-1*) |
| 19 | AGP31<br>*nhr-49(-); glp-1(-); Pnhr-49::nhr-49::GFP* | | | | | | | | | 81/88 | 26.6 ± 0.4 | <0.0001 | <0.0001 (*vs. glp-1*)<br><0.0001 (*vs. nhr-49; glp-1*)<br><0.0001 (*vs.* GFP-ve siblings) |
| 20 | AGP31 GFP-ve siblings | | | | | | | | | 91/92 | 14.9 ± 0.1 | <0.0001 | 0.0008 (*vs. nhr-49; glp-1*) |
