# Supplementary material for: Germline Signals Deploy NHR-49 to Modulate Fatty-Acid β-Oxidation and Desaturation in Somatic Tissues of C. elegans
Source: PLoS Genet. 2014 Dec 4;10(12):e1004829. doi: 10.1371/journal.pgen.1004829 (PMC4256272; doi:10.1371/journal.pgen.1004829)
Supplement: Table S4 — Effect of reduced IIS signaling and mitochondrial electron transport chain activity on the lifespan of nhr-49 mutants. S4A: Effect of nhr-49 mutation on daf-2 mutants' longevity. S4B: RNAi lifespans. Wild-type worms (N2) and nhr-49 mutants were subjected to whole-life RNAi inactivation of daf-2 (that encodes the insulin/IGF1 receptor) and genes involved in mitochondrial electron transport chain activity (cco-1 and cyc-1). See methods section for experimental details. In both tables, data is represented as mean lifespan in days (Mean) ± standard error of the mean (SEM). ‘n’ refers to the number of worms observed (obs) divided by total number of worms tested in the experiment. a some worms were censored from the analysis as described in methods. b control refers to worms exposed to empty vector plasmid without an RNAi insert. P values were calculated using the log rank (Mantel Cox) method. (PDF) [file pgen.1004829.s016.pdf]

Ratnappan et al., REVISED Table S4: Effect of *nhr-49* mutation on the longevity mediated by reduced IIS pathway activity and impaired mitochondrial electron transport

S4A: Genetic Mutants

		Trial 1					Trial 2					Trial 3				
#	Strain	n = 0bs/Total[a]	Mean ± SEM	P (*vs.* N2)	P (vs…)	% Lifespan Extension	n = 0bs/Total[a]	Mean ± SEM	P (*vs.* N2)	P (vs…)	% Lifespan Extension	n = 0bs/Total[a]	Mean ± SEM	P (*vs.* N2)	P (vs…)	% Lifespan Extension
1	N2	48/80	16.9 ± 0.1				22/55	22.5 ± 0.3				59/70	17.9 ± 0.5			
2	*nhr-49(nr2041)*	80/91	10.7 ± 0.1	<0.0001		- 37	43/57	12.2 ± 0.07	<0.0001		- 46	74/86	9.5 ± 0.2	<0.0001		- 47
4	*daf-2(e1370)*	42/67	43.0 ± 0.6	<0.0001		+ 154	51/89	43.8 ± 0.5	<0.0001		+ 97	80/92	40.6 ± 1.1	<0.0001		+ 126
5	*nhr-49(nr2041);daf-2(e1370)*	46/74	42.4 ± 0.6	<0.0001	0.004 {*vs. daf-2 (e1370)*}	+ 150	21/82	49.2 ± 1.4		0.0004 {*vs. daf-2 (e1370)*}	+ 118	70/90	34.3 ± 1.1	<0.0001	0.0001 {*vs. daf-2 (e1370)*}	+ 92
6	*daf-2(e1368)*	31/75	30.6 ± 0.4	<0.0001		+ 81	50/80	28.8 ± 0.6	<0.0001		+ 28	56/75	24.4 ± 1.1	<0.0001		+ 36
7	*nhr-49(nr2041);daf-2(e1368)*	45/86	29.5 ± 0.7	<0.0001	0.73 {*vs. daf-2 (e1368)*}	+ 74	63/94	26.8 ± 0.9	<0.0001	0.88 {*vs. daf-2 (e1368)*}	+ 19	46/64	21.2 ± 0.8	<0.0001	0.001 {*vs. daf-2 (e1368)*}	+ 18

S4B: RNAi inactivation

		Trial 1					Trial 2				
#	Strain (RNAi Treatment)	n = 0bs/Total[a]	Mean ± SEM	P (*vs.* Control[b])	P (*vs.*)	% Lifespan Extension	n = 0bs/Total[a]	Mean ± SEM	P (*vs.* Control[b])	P (*vs.*)	% Lifespan Extension
1	N2 (Control)	72/86	17.0 ± 0.2				81/90	16.9 ± 0.3			
2	N2 *(daf-2)*	58/84	34.6 ± 0.6	<0.0001		+ 50	70/90	27.9 ± 0.6	<0.0001		+ 40
3	N2 *(cyc-1)*	64/83	30.7 ± 1.1	0.0002		+ 45	85/90	19.6 ± 0.3	0.0002		+ 14
4	N2 *(cco-1)*	33/80	28.3 ± 0.3	<0.0001	P vs {*N2 (cyc-1)* 0.4	+ 40	81/90	21.0 ± 0.2	<0.0001	P vs {*N2 (cyc-1)* 0.17	+ 19
5	*nhr-49* (Control)	86/89	12.9 ± 0.2	<0.0001	P *vs* N2 (Control) <0.0001		86/89	11.6 ± 0.1	<0.0001	P *vs* N2 (Control) <0.0001	
6	*nhr-49 (daf-2)*	61/86	25.0 ± 0.4	<0.0001	P *vs* N2 (Control) <0.0001	+ 49	72/90	28.0 ± 0.3	<0.0001	P *vs* N2 (Control) <0.0001; P *vs* N2*(daf-2)* 0.33	+ 58
7	*nhr-49 (cyc-1)*	85/95	14.8 ± 0.2	0.0004		+ 13	85/95	14.8 ± 0.2	<0.0001		+ 22
8	*nhr-49 (cco-1)*	57/70	19.4 ± 0.2	<0.0001	P *vs nhr-49 (cyc-1)* <0.0001	+ 33	88/90	14.1 ± 0.2	<0.0001	P *vs nhr-49 (cyc-1)* 0.05	+ 18
