# Supplementary material for: Germline Signals Deploy NHR-49 to Modulate Fatty-Acid β-Oxidation and Desaturation in Somatic Tissues of C. elegans
Source: PLoS Genet. 2014 Dec 4;10(12):e1004829. doi: 10.1371/journal.pgen.1004829 (PMC4256272; doi:10.1371/journal.pgen.1004829)
Supplement: Table S5 — Effect of daf-16 mutation on the longevity of NHR-49::GFP worms. S5A: daf-16 mutants do not exhibit lifespan extension upon NHR-49 overexpression. S5B: NHR-49::GFP does not rescue the longevity of daf-16;glp-1 mutants. Data are represented as mean lifespan in days (Mean) ± standard error of the mean (SEM). ‘n’ refers to the number of worms observed (obs) divided by total number of worms tested in the experiment. a some worms were censored from the analysis as described in methods. P values were calculated using the log rank (Mantel Cox) method. (PDF) [file pgen.1004829.s017.pdf]

Ratnappan et al., REVISED Table S5: Effect of *daf-16* mutation on the longevity conferred by NHR-49 overexpression

		Trial 1					Trial 2				
**#**	**Strain**	**n = 0bs/Total[a]**	**Mean + SEM**	**P (*vs.* N2)**	**P (vs.**	**% Lifespan Change**	**n = 0bs/Total[a]**	**Mean + SEM**	**P (*vs.* N2)**		**% Lifespan Change**
**S5A: *daf-16* mutant background**											
1	N2	67/109	18.5 + 0.3				106/24	18.6 + 0.3			
2	*nhr-49(nr2041)*	87/96	12.7 + 0.1	<0.0001		-31	116/118	11.2 + 0.1	<0.0001		-40
3	*daf-16(mu86)*	56/120	15.6 + 0.2	0.001		-16	62/122	15.6 + 0.2	0.001		-16
4	*Pnhr-49::nhr-49::GFP*	40/98	24.9 + 0.2	<0.0001	<0.0001 (P *vs daf-16*, *daf-16;Pnhr-49::nhr-49::GFP* and *nhr-49*)	+ 34	37/115	24.1 + 0.1	<0.0001	<0.0001 (P *vs daf-16*, *daf-16;Pnhr-49::nhr-49::GFP* and *nhr-49*)	+ 29
5	*daf-16(mu86); Pnhr-49::nhr-49::GFP*	56/96	17.4 + 0.3	0.26	0.11 (*vs. daf-16*)	- 6	82/118	16.3 + 0.2	0.001	0.03 (*vs. daf-16*)	-12
**S5B: *daf-16;glp-1* mutant background**											
1	*glp-1*	75/91	36.9 + 0.2				72/80	31.6 + 0.3			
2	*nhr-49;glp-1*	76/100	14.2 + 0.2	<0.0001 (*vs. glp-1*)			47/64	15.7 + 0.3	<0.0001 (*vs. glp-1*)		
3	*daf-16;glp-1*	75/90	20.4 + 0.3	<0.0001 (*vs. glp-1*)			70/80	17.6 + 0.3	<0.0001 (*vs. glp-1*)		
4	*daf-16;glp-1;NHR-49::GFP*	74/89	22.8 + 0.3	<0.0001 (*vs. glp-1*)	0.001 (*P vs. daf-16;glp-1*); 0.16 (*vs.* non-transgenic sibling)		74/91	20.1 + 0.2	<0.0001 (*vs. glp-1*)	0.001 (*P vs. daf-16;glp-1*); 0.002 (*vs.* non-transgenic sibling)	
5	*daf-16;glp-1;NHR-49::GFP* non-transgenic siblings	80/90	20.2 + 0.2	<0.0001 (*vs. glp-1*)	0.17 (*vs. daf-16;glp-1*)		63/80	22.3 + 0.5	<0.0001 (*vs. glp-1*)	<0.0001 (*vs. daf-16;glp-1*)	
