# Supplementary material for: Germline Signals Deploy NHR-49 to Modulate Fatty-Acid β-Oxidation and Desaturation in Somatic Tissues of C. elegans
Source: PLoS Genet. 2014 Dec 4;10(12):e1004829. doi: 10.1371/journal.pgen.1004829 (PMC4256272; doi:10.1371/journal.pgen.1004829)
Supplement: Table S6 — Effect of RNAi inactivation of β-oxidation genes on the longevity of glp-1 mutants and wild-type worms. glp-1 mutants (1A) and the sterile strain, *fer-15(b26);fem-1(hc17), (1B) were subjected to ‘adult-only’ RNAi inactivation of mitochondrial β-oxidation genes. fer-15(b26);fem-1(hc17) is a temperature sensitive strain that when grown at 25°C is sterile and used as a surrogate for wild-type, N2 in lifespan assays. Data is shown as mean lifespan in days (Mean) ± standard error of the mean (SEM). ‘n’ refers to the number of worms observed (obs) divided by total number of worms tested in the experiment. a some worms were censored from the analysis as described in methods. b Empty vector control refers to worms exposed to empty vector plasmid without an RNAi insert. P values were calculated using the log rank (Mantel Cox) method. (PDF) [file pgen.1004829.s018.pdf]

Ratnappan et al., REVISED. Table S6: Mitochondrial fatty-acid β-oxidation genes regulated by NHR-49 are required for the increased longevity of germline-less animals

			S6A: RNAi Inactivation in Germline-Less Adults					S6B: RNAi Inactivation in Wild-Type* Adults			
**#**	**Enzyme Encoded**	**Gene**	**Cosmid**	**n = obs/total[b]**	**Mean ± SD**	**P (vs Control[b])**	**Percent Effect on Lifespan**	**n = obs/total[b]**	**Mean ± SD**	**P (vs Control[b])**	**Percent Effect on Lifespan**
		Empty Vector Control[a]		57/67	26.02 ± 0.95	0.00	0	64/65	15.06 ± 0.52	0.00	
		daf-16	R13H8.1	72/73	16.14 ± 0.52	0.00	- 38				
		tcer-1	ZK1127.9	62/65	18.75 ± 0.65	0.00	- 28				
		daf-2	Y55D5A.5					65/69	19.41 ± 0.8	0.00	+ 29
1	Acyl CoA Synthetase	*acs-2*	F28F8.2	62/63	22.52 ± 0.99	0.0216	- 13	78/80	13.8 ± 0.34	0.03	-8.00
2	Acyl CoA Synthetase	*acs-17*	C46F4.2	61/63	19.38 ± 0.75	<0.0001	- 26	65/72	13.78 ± 0.45	0.09	-8.00
3	Acyl CoA Synthetase	*acs-22*	D1009.1					54/54	12.98 ± 0.48	0.01	-14.00
4	Carnitine Palmiloyl Transferase	*cpt-2*	R07H5.2	43/45	23.01 ± 0.91	0.0027	- 12	59/62	14.22 ± 0.31	0.13	-5.00
5	Acyl CoA Dehydrogenase	*acdh-11*	Y45F3A.3	57/60	22.05 ± 0.91	0.0015	- 15	37/37	13.38 ± 0.63	0.05	-11.00
6	Enoyl CoA Hydratase	*ech-7*	Y105E8A.4	68/69	20.47 ± 0.88	0.0001	- 21	54/59	14.34 ± 0.58	0.44	-5.00
7	Hydroxyl Acyl CoA Dehydrogenase	*hacd-1*	R09B5.6	67/67	21.00 ± 0.92	0.0008	- 19	64/69	15.26 ± 0.55	0.65	ne
8	HADHA	*ech-1.2*	T08B2.7	65/66	22.39 ± 0.92	0.0055	- 14	40/40	13.85 ± 0.71	0.28	-8.00
9	Thiolase	*acaa-2*	F53A2.7	63/64	23.97 ± 0.92	0.06	- 8	58/62	13.94 ± 0.63	0.35	-7.00
