# Supplementary material for: Germline Signals Deploy NHR-49 to Modulate Fatty-Acid β-Oxidation and Desaturation in Somatic Tissues of C. elegans
Source: PLoS Genet. 2014 Dec 4;10(12):e1004829. doi: 10.1371/journal.pgen.1004829 (PMC4256272; doi:10.1371/journal.pgen.1004829)
Supplement: Table S7 — Oleic acid supplementation does not rescue the lifespan phenotypes of nhr-49 mutants. (PDF) [file pgen.1004829.s019.pdf]

Ratnappan et al., REVISED. Table S7. Oleic Acid supplementation does not rescue lifespan phenotypes of *nhr-49* mutants				
**Strain (Supplement)**	**n = Obs[a]/Total**	**Mean + SEM**	**P (OA *vs.* Unsupplemented)**	**P (*vs.* Internal Controls)**
N2 (OA)	48/60	19.8 + 0.8	0.0141 (*vs.* N2)	
nhr-49 (OA)	43/75	11.0 + 0.3	<0.0001 (*vs. nhr-49)*	<0.0001 [*vs.* N2 (OA), *glp-1 (OA)]*
glp-1 (OA)	43/60	30.8 + 1.1	0.0093 (*vs. glp-1)*	<0.0001 [*vs.* N2 (OA), *nhr-49* (OA) and *nhr-49;glp-1* (OA)]
nhr-49;glp-1 (OA)	65/75	9.2 + 0.1	<0.0001 (*vs. nhr-49;glp-1)*	<0.0001 [*vs.* N2 (OA), *glp-1* (OA)]
N2	29/38	23.32 + 1.11		
nhr-49	73/75	9.01 + 0.23		<0.0001 (*vs.* N2 and *glp-1*)
glp-1	54/61	33.84 + 1.02		<0.0001 (*vs.* N2, *nhr-49* and *nhr-49;glp-1*)
nhr-49;glp-1	74/75	12.00 + 0.21		<0.0001 (*vs.* N2, *glp-1*)

OA: Oleic Acid

[a] Number of worms observed in the experiment
