# Supplementary material for: Germline Signals Deploy NHR-49 to Modulate Fatty-Acid β-Oxidation and Desaturation in Somatic Tissues of C. elegans
Source: PLoS Genet. 2014 Dec 4;10(12):e1004829. doi: 10.1371/journal.pgen.1004829 (PMC4256272; doi:10.1371/journal.pgen.1004829)
Supplement: Table S8 — List of strains used in this study. (PDF) [file pgen.1004829.s020.pdf]

Ratn *et al., REVISED.* Table S8: Strains used in this study		
**Strain**	**Genotype**	**Comments**
N2	Wild-type	
CF1903	*glp-1(e2141ts) III*	
CF1880	*daf-16(mu86) I;glp-1(e2141ts) III*	
CF2154	*tcer-1(tm1452) II;glp-1(e2141ts) III*	
CF1038	*daf-16(mu86) I*	
CF1041	*daf-2(e1370) III*	
DR1572	*daf-2(e1368) III*	
CF512	*fer-15(b26) II; fem-1(hc/7) IV*	
CF2573	*glp-1(e2141ts) III;sIs10314(Pdod-8::gfp + pCeh361)*	
AGP12a	*nhr-49(nr2041) I*	obtained by out-crossing 3x to Ghazi lab N2
AGP22	*nhr-49(nr2041) I;glp-1(e2141ts) III*	obtained by crossing AGP12a to CF1903
AGP94	*nhr-49(nr2041) I;daf-2(e1370) III*	obtained by crossing AGP12a to CF1041
AGP95	*nhr-49(nr2041) I;daf-2(e1368) III*	obtained by crossing AGP12a to DR1572
AGP24f	*glmEx5 (Pnhr-49::nhr-49::GFP + Pmyo-2::mCherry) (NHR-49::GFP)*	Injected pAG4 and *Pmyo-2::mCherry (*at 100 ng/μl and 15 ng/μl, respectively) into N2
AGP28c	*glmEx6 (Pnhr-49::nhr-49::GFP + Pmyo-2::mCherry)*	Injected pAG4 and *Pmyo-2::mCherry (*at 25 ng/μl and 3.75 ng/μl, respectively) into N2
AGP25	*glp-1(e2141ts) III;glmEx5 (Pnhr-49::nhr-49::GFP + Pmyo-2::mCherry)*	obtained by crossing AGP24f to CF1903
AGP29a	*glp-1(e2141ts) III;glmEx7 (Pnhr-49::nhr-49::GFP + Pmyo-2::mCherry)*	Injected pAG4 and *Pmyo-2::mCherry (*at 25 ng/μl and 3.75 ng/μl, respectively) into CF1903
AGP33a	*nhr-49(nr2041) I;glmEx8 (Pnhr-49::nhr-49::GFP + Pmyo-2::mCherry)*	Injected pAG4 and *Pmyo-2::mCherry (*at 100 ng/μl and 15 ng/μl, respectively) into AGP12a
AGP30b	*nhr-49(nr20410 I;glmEx9 (Pnhr-49::nhr-49::GFP + Pmyo-2::mCherry)*	Injected pAG4 and *Pmyo-2::mCherry (*at 25 ng/μl and 3.75 ng/μl, respectively) into AGP12a
AGP34a	*nhr-49(nr2014) I;glp-1(e2141ts) III; glmEx10 (Pnhr-49::nhr-49::GFP + Pmyo-2::mCherry)*	Injected pAG4 and *Pmyo-2::mCherry (*at 100 ng/μl and 15 ng/μl, respectively) into AGP22
AGP31	*nhr-49(nr2014) I;glp-1(e2141ts) III; glmEx11 (Pnhr-49::nhr-49::GFP + Pmyo-2::mCherry)*	Injected pAG4 and *Pmyo-2::mCherry (*at 25 ng/μl and 3.75 ng/μl, respectively) into AGP22
AGP109	*daf-16(mu86)I;glmEx24[Pnhr-49::nhr-49::GFP + pmyo-2::Cherry]*	Injected pAG4 and *Pmyo-2::mCherry (*at 100 ng/μl and 15 ng/μl, respectively) into CF1038
