# Supplementary material for: Germline Signals Deploy NHR-49 to Modulate Fatty-Acid β-Oxidation and Desaturation in Somatic Tissues of C. elegans
Source: PLoS Genet. 2014 Dec 4;10(12):e1004829. doi: 10.1371/journal.pgen.1004829 (PMC4256272; doi:10.1371/journal.pgen.1004829)
Supplement: Table S9 — Q-PCR primers for genes examined in this study. (PDF) [file pgen.1004829.s021.pdf]

Ratn *et al.*, REVISED. Table S9: Q-PCR primers of genes examined in this study.

Gene	Cosmid	Primer sequence	
acs-2	F28F8.2	Forward Primer	5' tccggataaggagttctgtg 3'
		Reverse Primer	5' atttgacggacgtcatggt 3'
acs-17	C46F4.2	Forward Primer	5' tatgctcaggttgacaaacg 3'
		Reverse Primer	5' aataattggctccttctcctg 3'
acs-22	D1009.1	Forward Primer	5' ttgaatcaggtgaaacagca 3'
		Reverse Primer	5' caattcttgatacgtcaactgc 3'
cpt-2	R07H5.2	Forward Primer	5' gcaagatggattcggtattgg 3'
		Reverse Primer	5' tcacgtttcgacttattgctc 3'
acdh-11	Y45F3A.3	Forward Primer	5' aatacagccacgcaaagac 3'
		Reverse Primer	5' cgagaattgggtcatctttataagg 3'
ech-7	Y105E8A.4	Forward Primer	5' gagaatgactatttcacgaactgg 3'
		Reverse Primer	5' ctaaggcaaatccgttgacag 3'
hacd-1	R09B5.6	Forward Primer	5' atactgatattccgtccgctg 3'
		Reverse Primer	5' ggaagatgtcaagtttcagatcc 3'
ech-1.2	T08B2.7	Forward Primer	5' tggataagctccaatccga 3'
		Reverse Primer	5' atttggatgtcagctcctg 3'
acaa-2	F53A2.7	Forward Primer	5' atcttcgaggtaaatgaggc 3'
		Reverse Primer	5' ccaccattgacattcagct 3'
acdh-2	C17C3.12	Forward Primer	5' agtaatgcaccgattgctg 3'
		Reverse Primer	5' ccaagattatcatcctctgtatcc 3'
acdh-9	F28A10.6	Forward Primer	5' ggcagacttccagtataacc 3'
		Reverse Primer	5' gcgtttctaacgattagtctg 3'
ech-1.1	C29F3.1	Forward Primer	5' ctgaggctaaggcatttgg 3'
		Reverse Primer	5' cattagtcgatccttcgaacag 3'
cpt-5	F09F3.9	Forward Primer	5' cttctgagtaccagtaatattccc 3'
		Reverse Primer	5' accaccaagcattatcttgtc 3'
fat-5	W06D12.3	Forward Primer	5' cgagatccgacaaatgcagg 3'
		Reverse Primer	5' aacacaagctgatatagtccg 3'
fat-6	VZK822L.1	Forward Primer	5' cttctgtatgtgttcggagg 3'
		Reverse Primer	5' gatgacgtcattttgaagagc 3'
fat-7	F10D2.9	Forward Primer	5' gctctctatgtgttctcagg 3'
		Reverse Primer	5' caatgatgtcgttttgaagagc 3'
nhr-49	K10C3.6	Forward Primer	5' ttggcagaggtggattctc 3'
		Reverse Primer	5' ctgtaaagagaccggagcc 3'
pod-2	W09B6.1	Forward Primer	5' gagcagtattacgagacgc 3'
		Reverse Primer	5' gagctgctgttcatatcgt 3'
fasn-1	F32H2.5	Forward Primer	5' ggataatactggagaaggatcg 3'
		Reverse Primer	5' atgttgtccgaagactgag 3'
dgat-2	F59A1.10	Forward Primer	5' tcggatatcatcctcatgga 3'
		Reverse Primer	5' atgcctttgaacacttgct 3'
rpl-32	T24B8.1	Forward Primer	5' agggaattgataaccgtgtccgca 3'
		Reverse Primer	5' gtaggactgcatgaggagcatgt 3'
T7-primer			5' taatacgactcactataggg 3'
