# Supplementary material for: Germline Signals Deploy NHR-49 to Modulate Fatty-Acid β-Oxidation and Desaturation in Somatic Tissues of C. elegans
Source: PLoS Genet. 2014 Dec 4;10(12):e1004829. doi: 10.1371/journal.pgen.1004829 (PMC4256272; doi:10.1371/journal.pgen.1004829)
Supplement: Table S2 — Effect of RNAi inactivation of select ‘nhr’ RNAi clones on longevity of glp-1 mutants. glp-1 mutants were subjected to RNAi inactivation of ‘nhr’ genes found to be required for the up-regulation of Pstdh-1/dod-8::gfp following germline loss. See methods section for experimental details. Data from four independent trials are shown here, represented as mean lifespan in days (Mean) ± standard error of the mean (SEM). ‘n’ refers to the number of worms observed (obs) divided by total number of worms tested in the experiment. a some worms were censored from the analysis as described in methods. b Control (Ctrl) refers to worms exposed to control empty vector plasmid without an RNAi insert. P values were calculated using the log rank (Mantel Cox) method. (PDF) [file pgen.1004829.s014.pdf]

Ratnappan et al., REVISED. Table S2: Effect of RNAi inactivation of select suppressor 'nhr' clones on longevity of *glp-1* mutants

		Whole-Life RNAi Trial #1				Whole-Life RNAi Trial #2				Adult-Only RNAi Trial #1				Adult-Only RNAi Trial #2			
	RNAi Clone	Mean + SEM	P *vs.* Empty Vector Ctrl.[a]	n = obs/ total	% Suppression	Mean + SEM	P *vs.* Empty Vector Ctrl.[a]	n = obs/ total	% Suppression	Mean + SEM	P *vs.* Empty Vector Ctrl.[a]	n = obs /total	% Suppression	Mean + SEM	P *vs.* Empty Vector Ctrl.[a]	n = obs/ total	% Suppression
1	Empty Vector	26.2 + 0.5		84/89		19.1 + 0.2		51/62		27.5 + 0.2		70/77		26.3 + 0.6		92/96	
2	*daf-16*	14.3 + 0.1	<0.0001	73/90	45.4	11.4 + 0.2	<0.0001	83/90	40.3	14.5 + 0.2	<0.0001	84/87	47.2	16.4 + 0.2	<0.0001	92/97	37.6
3	*tcer-1*	17.0 + 0.2	<0.0001	82/86	35.1	15.1 + 0.5	<0.0001	49/78	20.9	21.8 + 0.5	<0.0001	82/85	20.7	22.3 + 0.3	0.0003	82/86	15.2
4	*daf-12*	17.1 + 0.3	<0.0001	85/97	34.7	15.7 + 0.2	<0.0001	66/91	17.8	19.7 + 0.3	<0.0001	69/88	28.3	24.7 + 0.4	0.012	98/97	6
5	*nhr-31*	17.0 + 0.3	<0.0001**	91/110	35.1	20.0 + 0.4	0.36	69/90	ne	21.8 + 0.6	<0.0001	87/95	20.7				
6	*nhr-49*	14.5 + 0.1	<0.0001*	99/103	44.6	10.8 + 0.05	<0.0001	61/90	43.4	14.3 + 0.2	<0.0001*	89/92	48	17.3 + 0.3	<0.0001*	99/101	34.2
7	*nhr-49 MV*	14.4 + 0.1	<0.0001*	63/97	45	9.8 + 0.3	<0.0001	89/90	48.6	14.3 + 0.3	<0.0001*	83/85	48	15.5 + 0.1	<0.0001*	79/92	41
8	*nhr-54*	20.2 + 0.3	0.0001	101/104	22.9	15.1 + 0.5	0.001	81/88	20.9	21.2 + 0.5	<0.0001	81/96	22.9	22.7 + 0.2	0.0026	100/104	13.6
9	*nhr-60*	21.3 + 0.9	0.0002	102/106	18.7												
10	*nhr-71*	22.7 + 0.3	0.0007	103/104	13.3												
11	*nhr-81*	19.4 + 0.4	<0.0001	75/99	25.9	15.8 + 0.6	0.01	74/98	17.2	23.6 + 0.4	0.007	66/90	14.1				
12	*nhr-87*	23.4 + 0.5	0.01	77/101	10.6												
13	*nhr-87 MV*	23.3 + 0.6	0.03	90/94	11												
14	*nhr-209*	21.6 + 0.7	0.0004	104/105	17.5												
15	*nhr-212*	17.2 + 0.2	<0.0001**	94/101	34.3	16.0 + 0.6	0.01	67/89	16.2	23.3 + 0.8	0.002	85/85	15.2				
16	*nhr-213*	21.5 + 0.6	0.002	95/96	17.9												
17	*nhr-255*	18.0 + 0.4	<0.0001**	109/109	31.2	18.1 + 0.4	0.38	71/90	ne	22.8 + 0.4	0.0001	88/101	17				
18	*nhr-274*	23.0 + 0.7	0.0031	98/100	12.2												
19	*nhr-274 MV*	23.1 + 0.4	0.004	100/101	11.8												
		P *vs daf-16* RNAi *0.94, 0.63				ne: no effect				P *vs daf-16* RNAi * 0.44, 0.83				P *vs daf-16* RNAi * 0.01, 0.005			
		0.06, 0.87															
